# Identification of novel gene signatures and immune cell infiltration in intervertebral disc degeneration using bioinformatics analysis

**DOI:** 10.3389/fmolb.2023.1169718

**Published:** 2023-07-14

**Authors:** Tao Tang, Zhongyuan He, Zhengya Zhu, Fuan Wang, Hongkun Chen, Fu Zhang, Jiaxiang Zhou, Jianmin Wang, Baoliang Li, Xizhe Liu, Zhiyu Zhou, Shaoyu Liu

**Affiliations:** ^1^ Innovation Platform of Regeneration and Repair of Spinal Cord and Nerve Injury, Department of Orthopaedic Surgery, The Seventh Affiliated Hospital, Sun Yat-sen University, Shenzhen, China; ^2^ Guangdong Provincial Key Laboratory of Orthopedics and Traumatology, The First Affiliated Hospital of Sun Yat-sen University, Guangzhou, China; ^3^ Department of Orthopaedic Surgery, The Affiliated Hospital of Qingdao University, Qingdao, China

**Keywords:** intervertebral disc degeneration, gene signatures, immune cells infiltration, druggene interaction analysis, bioinformatics analysis

## Abstract

**Background:** Intervertebral disc degeneration (IDD) is the leading cause of lower back pain, and an overall understanding of the molecular mechanisms related to IDD is still lacking. The purpose of this study was to explore gene signatures and immune cell infiltration related to IDD via bioinformatics analysis.

**Methods:** A total of five expression profiles of mRNA and non-coding RNA were downloaded from the Gene Expression Omnibus (GEO) database. The potentially involved lncRNA/circRNA–miRNA–mRNA networks and protein-protein interaction networks were constructed by miRNet, circBank, STRING, and the Cytoscape database. Gene ontology, Kyoto Encyclopaedia of Genes and Genomes Analysis, Gene Set Enrichment Analysis, Gene Set Variation Analysis, Immune Infiltration Analysis, and Drug-Gene Interaction were used to analyse the top 20 hub genes. RT-qPCR was conducted to confirm the 12 differential expressions of genes both in the nucleus pulposus and annulus fibrosus tissues

**Results:** There were 346 differentially expressed mRNAs, 12 differentially expressed miRNAs, 883 differentially expressed lncRNAs, and 916 differentially expressed circRNAs in the GEO database. Functional and enrichment analyses revealed hub genes associated with platelet activation, immune responses, focal adhesion, and PI3K-Akt signalling. The apoptotic pathway, the reactive oxygen species pathway, and oxidative phosphorylation play an essential role in IDD. Immune infiltration analysis demonstrated that the Treg cells had significant infiltration, and three levels of immune cells, including dendritic cells, Th2 cells, and tumour-infiltrating lymphocytes, were inhibited in IDD. Drug-gene interaction analysis showed that COL1A1 and COL1A2 were targeted by collagenase *clostridium* histolyticum, ocriplasmin, and PDGFRA was targeted by 66 drugs or molecular compounds. Finally, 24 cases of IDD tissues and 12 cases of normal disc tissues were collected, and the results of RT-qPCR were consistent with the bioinformatics results.

**Conclusion:** Our data indicated that the 20 hub genes and immune cell infiltration were involved in the pathological process of IDD. In addition, the PDGFRA and two potential drugs were found to be significant in IDD development.

## Introduction

Low back pain (LBP) is the most common musculoskeletal condition affecting adults worldwide (Wu et al., 2021). Intervertebral disc degeneration (IDD) has been regarded as the primary cause of LBP (Fontana et al., 2015). In the case of IDD, various etiologies have been implicated, like mechanical stress, injury, aging, and genetic factors (Guo H. Y. et al., 2020). At present, the clinical interventions for IDD mainly include drug therapy and surgery. However, these treatments only provide temporary painful relief and cannot provide permanent treatment (Liu Y. et al., 2019).

Recent research has shown that immunological infiltration plays a crucial role in the development and progression of IDD (Wang et al., 2021; Li et al., 2022; Zhao et al., 2022). Immune cells involvement with released inflammatory mediators and increased oxidative stress accelerate the inflammatory cascade (Lan et al., 2022). Besides, accumulated evidence reveals that mitochondrial-related genes are strictly linked to cell functioning and modulate cell immune activity in response to damaged associated signals from nucleus pulposus cells (NPCs). So, finding genes linked to mitochondria could lead to new ways to diagnose and treat IDD (Guo et al., 2023).

The targeted hub genes for potential drugs have been explored in the procession of IDD (Chen et al., 2020). IDD is protected from by the phosphatidylinositol 3-kinase (PI3K)/Akt pathway, which is linked to an increase in extracellular matrix (ECM) content (Ouyang et al., 2017). According to a disease-gene interaction network, some medicines may have curative benefits on IDD (Zhan et al., 2021). However, there are currently few targeted IDD medications.

The study compared hub gene expression in nucleus pulposus (NP) and annulus fibrosus (AF) tissues between healthy individuals and IDD patients from the Gene Expression Omnibus (GEO) datasets. The protein-protein interaction (PPI) and lncrna/circrNA-miRNA-mRNA regulatory networks were constructed using public datasets. Hub genes’ roles in IDD were explored through Gene Ontologies (GOs), Kyoto Encyclopedia of Genes and Genomes (KEGG), immune infiltration analysis, and hub gene-drug interaction network. The study aimed to elucidate IDD pathophysiology, identify biomarkers and determine the role of immune infiltration in IDD.

## Materials and methods

### Gene expression data collection

The gene expression profiles of IDD, including GSE15227, GSE23130, GSE19943, GSE116726, and GSE153761, were downloaded from GEO databases (http://www.ncbi.nlm.nih.gov/geo). Three unhealthy disc tissues and twelve healthy discs were included in the GSE15227 dataset. Eight degenerative disc tissues and fifteen controls were included in the GSE23130 dataset. Three controls and three degenerative NPCs were included in the GSE19943 dataset. The GSE116726 dataset included three NP tissues from IDD patients and three controls from fresh traumatic lumbar fracture patients. The GSE153761 dataset included three degenerative cervical cartilage endplates and three controls (Supplementary Table S1).

### Differentially expressed genes

First, we performed data normalisation. The lists of differentially expressed genes (DEGs), including differentially expressed mRNAs (DE mRNAs), miRNAs (DE miRNAs), lncRNAs (DE lncRNAs), and circRNAs (DE cirRNAs) between control and IDD group, were created using the “limma” algorithm (Ritchie et al., 2015). The thresholds were absolute value of log2 (fold change) (log2FC) > 1 and *p* < 0.05. The genes with log2FC > 1 with an adjusted *p* < 0.05 were upregulated, and the genes with log2FC < −1 with an adjusted *p* < 0.05 were downregulated. *p* values under 0.05 and false discovery rates (FDR) *p*-value of less than 0.20 were considered significant. The ggplot2 program in the R software was then used to draw the heatmap and volcano plot. The Venn diagram was generated with a publicly available online tool (http://bioinformatics.psb.ugent.be/webtools/Venn/).

### Construct the ceRNA regulatory network

We obtained the DE miRNAs related mRNAs and lncRNAs from the miRNet database (Fan and Xia, 2018) and the DE miRNAs related mRNAs and cirRNAs from circbank database (Liu M. et al., 2019) and visualized the lncRNA/circRNA-miRNA–mRNA regulatory network using Cytoscape software (Shannon et al., 2003).

### PPI network construction

The Search Tool for the Retrieval of Interacting Genes/Proteins (STRING) database (Szklarczyk et al., 2019) searches for interactions between verified and predicted proteins. The PPI network was built using the STRING database. The top 20 genes in the PPI network with the highest molecular complex detection (MCODE) scores (Bandettini et al., 2012) were designated as hub genes.

### Functional enrichment analyses of DEGs

GO analysis is a common method for large-scale functional enrichment studies. GO may be categorized into three categories: biological process (BP), molecular function (MF), and cellular component (CC) (Yu, 2020). The KEGG is an extensive database that combines data on genomic, chemical, and system functional information (Kanehisa and Goto, 2000). The role of DEGs was examined using the “cluster Profiler” of the R package (Yu et al., 2012).

The biological process of the GO was enriched using the Gene Set Enrichment Analysis (GSEA) tool (Subramanian et al., 2005). According to the grading of the phenotypic correlation degree, the mRNAs in all datasets were separated into high- and low expression groups. All DEGs in the two groups were then enriched and evaluated using the cluster Profiler package. Reference gene sets from the Molecular Signatures Database of c2 (c2. cp.v7.2. symbols) (Liberzon et al., 2015). *p* values under 0.05 and false discovery rates (FDR) *p* -value of less than 0.20 were considered significant.

The R package Gene Set Variation Analysis (GSVA) was used to investigate BP and KEGG pathways of DEGs between different groups (Hänzelmann et al., 2013). The enrichment scores were computed for each sample in each gene set, with the lowest gene set being 5 and the highest gene set being 5,000. The final enrichment score matrix was obtained. The limma software was used to compare the variations in GSVA scores between the hub genes in the two data sets. *p* values under 0.05 and an FDR *p*-value of less than 0.20 were regarded as significant.

### Immune infiltration related analysis

In order to assess the immune infiltration microenvironment, the single-sample gene-set enrichment analysis (ssGSEA) method was used to compare the relative abundance of 16 types of immune cells and 13 immune-related pathways in IDD patients and controls. The relative abundance of each immune cell infiltration in each sample was shown by the enrichment scores determined by the ssGSEA analysis (Barbie et al., 2009; Charoentong et al., 2017). The ggplot2 software was used to show the association between immune cell expression differences in the mRNA dataset.

### Construct mRNA-RBP, mRNA-TF, mRNA-drugs interaction networks

The targeting connection between mRNA and RNA-binding protein was predicted using the Starbase v2.0 database for RNA-binding protein (RBP) (http://starbase.sysu.edu.cn/). Predictions of potential drugs or small molecule compounds that interact with hub genes were made using the DGidb database (https://www.dgidb.org) (Freshour et al., 2021). Transcriptional factors (TFs) associated with hub genes were found in the transcriptional regulatory relationships unraveled by sentence-based text mining (TRRUST) database (https://www.grnpedia.org/trrust/). The mRNA-RBP, mRNA-drugs, and mRNA-TF interaction networks were shown using Cytoscape.

### Random forest and receiver operating characteristic curve analysis

The Random forest (RF) an ensemble-learning approach for classification that employs a number of decision trees, each of which is composed of a random selection of data (Gruber et al., 2012). We used the “randomForest” R package (Liu and Zhao, 2017) to perform random forest analysis on the expression levels of hub genes in the GSE15227 and GSE23130 datasets. The parameters were set as set. seed (234) and ntree = 1000. Then, we screened the specific analysis results of hub genes using increase in node purity (IncNodePurity) > 0.1 as the criterion.

Receiver operating characteristic (ROC) is a graphical analysis tool that can be used to select the best model, discard the second-best model, or set the best threshold in the same model (Mandrekar, 2010). The ROC curve is a comprehensive indicator of continuous variables reflecting sensitivity and specificity, and the correlation between sensitivity and specificity is reflected by the composition method. The R “pROC” package was used to draw the ROC curves of hub genes between different groups in the GSE15227 and GSE23130 dataset, and the area under curve (AUC) was calculated to evaluate the diagnostic effect of hub genes on IDD.

### Extraction and culture of primary intervertebral disc cells

NP and AF tissues were digested at 37°C with 2 mg/ml type II collagenase (Gibco, United States). After washing with phosphate buffered saline (PBS), the digested tissues were placed in an incubator at 5% CO_2_ and 37°C with Dulbecco’s Modified Eagle Medium (DMEM) (Gibco, United States) containing 10% fetal bovine serum (Gibco, United States) and 1% penicillin/streptomycin (Gibco, United States). The cells at the confluent stage were passaged following digestion with 0.25% trypsin-ethylene diamine tetraacetic acid (EDTA) (Gibco, United States). Cells after the three passages were used in the following experiments.

### Patients and ethics

The ethics permissions were supplied by the institutional review board of the Seventh Affiliated Hospital of Sun Yat-sen University (KY-2021-030-01). Each patient who participated in the study gave their informed written permission. Magnetic resonance imaging (MRI) was used in accordance with the Pfirrmann classification to assess the severity of IDD (Pfirrmann et al., 2001). All MRI were performed with a 3.0-T magnetic resonance scanner (General Electric Company, PHILIPS Ingenia Elition). T1-weighted images are helpful in identifying the anatomy of the lumbar spine and differentiating between different tissues such as bones, muscles, and discs. T2-weighted images are highly sensitive to water content and can help identify a herniated disc by showing the abnormal protrusion of the disc material into the spinal canal. Pfirrmann I-II tissue samples were utilized as controls. Human lumbar disc tissues were acquired from patients who had spinal canal decompression therapy.

### Real-time quantitative polymerase chain reaction (RT-qPCR)

RNA was directly extracted from tissues after separating the NP and AF tissues in accordance with a prior methodology (Caprez et al., 2018). In brief, a sample weighing 150 mg was divided, digested at 37 °C with 2 mg/ml pronase, flash-frozen, pulverized in liquid nitrogen, and homogenized with a tissue lyser (Qiagen/Retsch^®^, Germany). A TRI Reagent (Invitrogen, United States) was used to extract the total RNA, and 400 ng of RNA was converted to cDNA using a cDNA synthesis kit (Takara, Japan). For reverse transcription: we first measured the sample RNA concentration in a spectrophotometer (NanoDrop™ One/2000, United States) and calculated the required volume of 400 ng RNA. Then 400 ng of RNA, 4 μL of mix, and DEPC water (Beyotime, China) were mixed to make a 20 μL mixture solution. Finally, the mixture was reverse transcribed in a PCR instrument (Bio-Rad, United States). RT-qPCR was performed using qPCR Mix (Thermo Fisher Scientific, United States) in a CXF-96 real-time system (Bio-Rad, United States). The reaction mixture contained the following components in a total volume of 10 µL: 5 µL Fast SYBR Green (Thermo Fisher Scientific, United States) master mix, 2 μL RNase-free (Beyotime, China) ddH_2_O, 2 μL cDNA and 0.5 μL of each primer (Supplementary Table S2). Relative expression was calculated using the 2^−ΔΔCt^ and gene expression levels were normalized to GAPDH.

### Immunohistochemistry

Intervertebral disc tissues were obtained immediately after surgery. IDD specimens were fixed in 4% paraformaldehyde and then embedded in paraffin. Tissue sections (5 μm) were deparaffinized, rehydrated, and incubated overnight at 4°C with primary antibodies. After washing with PBS, the sections were incubated with a horseradish peroxidase-conjugated secondary antibody. Then, the reaction was developed with 3,3′-diaminobenzidine and counterstained with haematoxylin. The relative protein expression was quantified by Image-Pro Plus version 6.0 software (Media Cybernetics Inc.). The following primary antibodies were used: anti-ACTG1 polyclonal antibody (solarbio, 1:200) and anti-CALM3 polyclonal antibody (affinity, 1:100).

### Statistical analysis

All data were analyzed and processed on R software (version 4.1.2) and all results are provided as mean ± standard deviation (SD). The “pROC” package was used to calculate the AUC and draw the ROC curves. The Shapiro–Wilk normality test was performed to evaluate the normality of the data distribution. Statistical significance (*p* < 0.05) was analyzed by Student’s *t-*test (two groups) or one-way analysis of variance (ANOVA) (more than two groups). A Mann-Whitney U test was performed for non-normally distributed data.

## Results

### Identification of DEGs related to IDD

**FIGURE 1:**
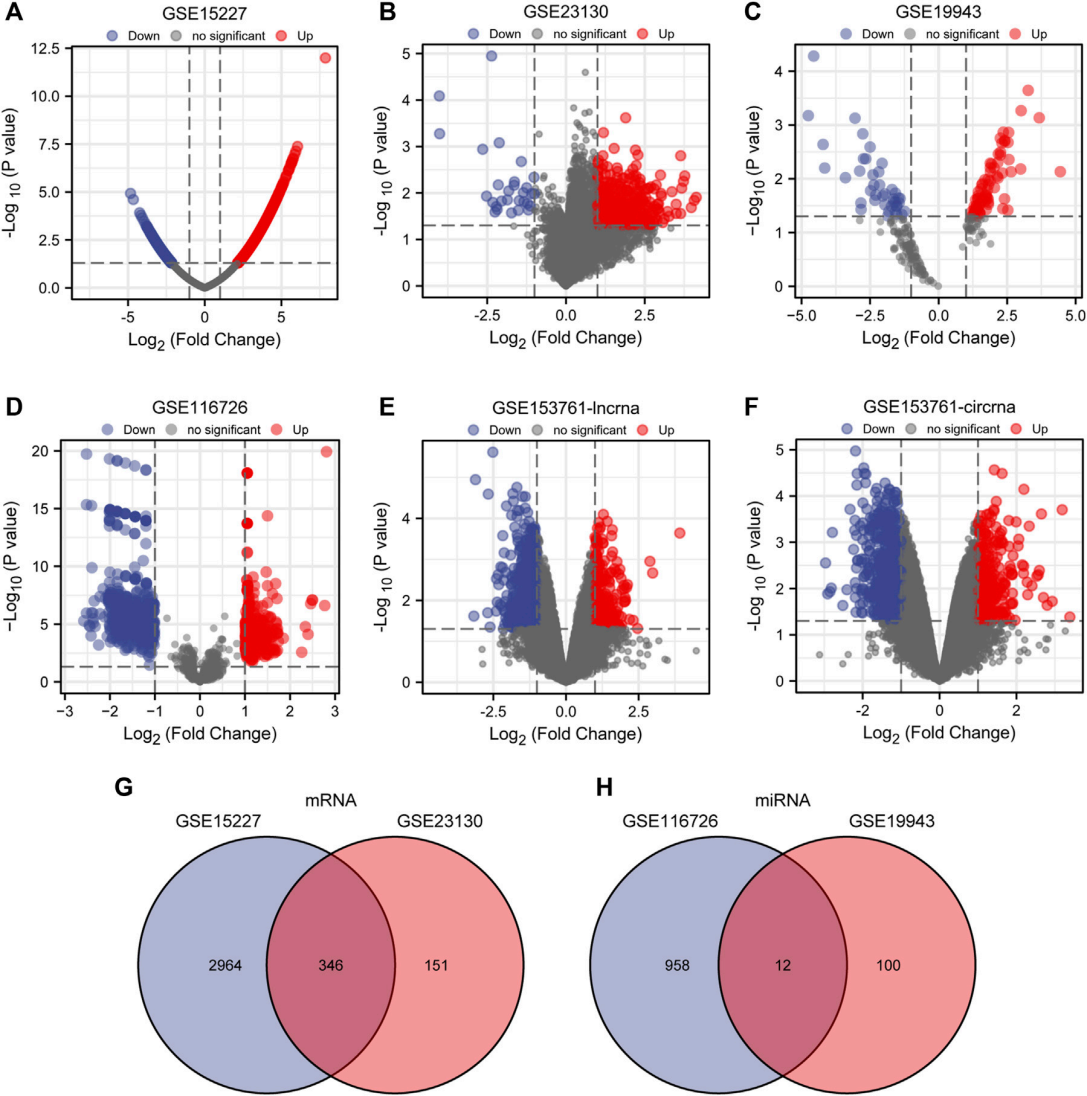
Differential expression of mRNA, miRNA, lncRNA and cirRNA associated with IDD. **(A–F)** Volcano map of DE mRNAs, miRNAs, lncRNAs, and cirRNAs in GSE15227, GSE23130, GSE19943, GSE116726, and GSE153761 between IDD patients and healthy controls. Genes that have been upregulated are shown in red, those that have been downregulated in blue, and unaltered genes are represented in black. **(G, H)** DE miRNAs between GSE116726 and GSE19943 and DE mRNAs between GSE15227 and GSE23130 are shown in a Venn diagram.

**FIGURE 2:**
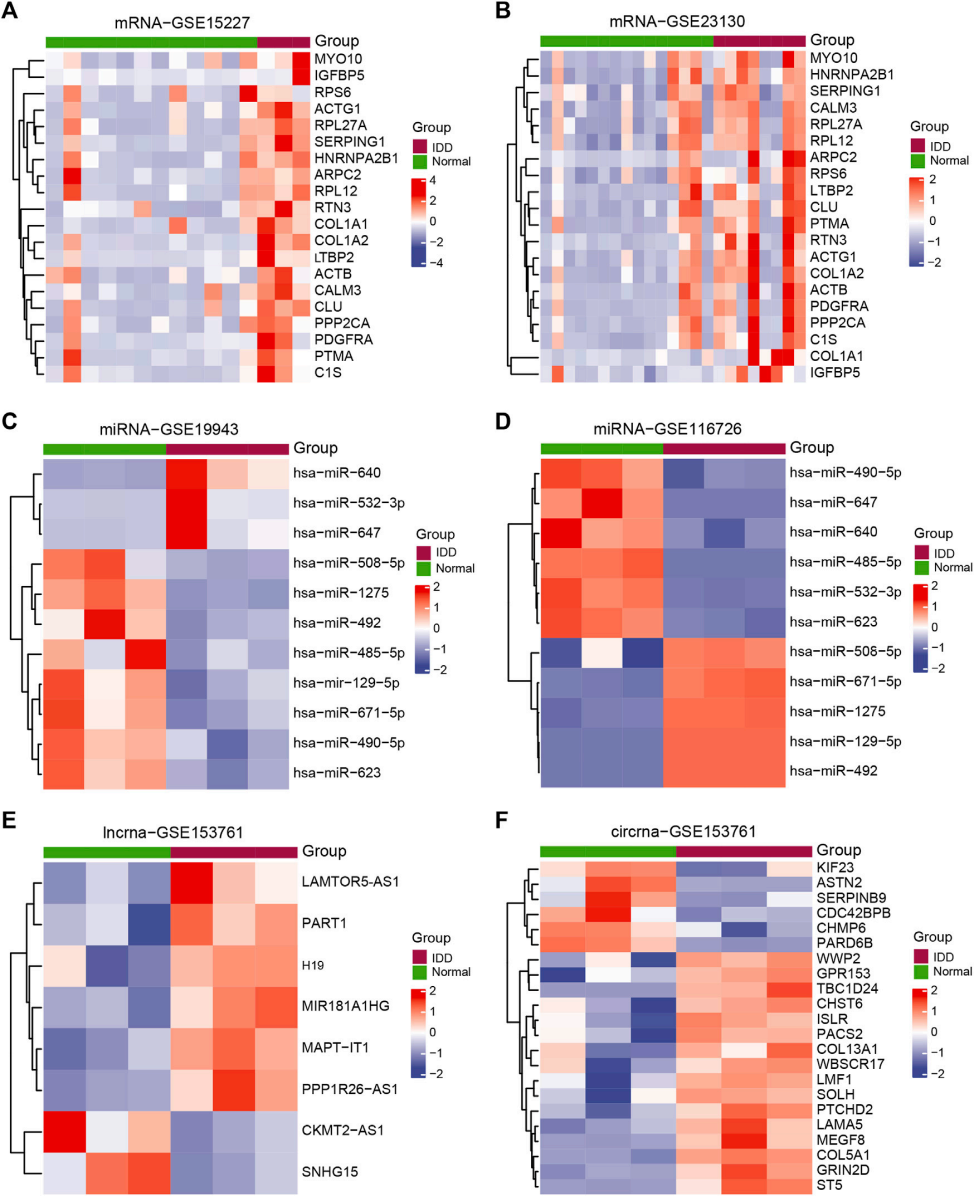
Exhibits the DEGs related to IDD. Heatmap of DEGs between IDD patients and healthy controls in **(A)** GSE15227, **(B)** GSE23130, **(C)** GSE19943, **(D)** GSE116726, and **(E–F)** GSE153761. Patients with IDD are shown in red, whereas healthy controls are shown in green.

Batch correction, normalization, and difference analysis of RNA-seq data from GSE15227, GSE23130, GSE19943, GSE116726, and GSE153761 were carried out to check for DEGs in IVD samples. In GSE15227 datasets, a total of 3,314 DE mRNAs were found, including 1,740 upregulated and 1,574 downregulated mRNAs, and in GSE23130 datasets, 497 DE mRNAs were found, including 468 upregulated and 29 downregulated mRNAs. Volcanic plots, a Venn diagram, and a heat map were used to display the findings (Figures 1A, B, G, Figures 2A, B; Supplementary Table S3). A total of 112 DE miRNAs, comprising 71 upregulated and 41 downregulated miRNAs, and 970 DE miRNAs, including 449 upregulated and 521 downregulated miRNAs, were found in the GSE19943 and GSE116726 datasets, respectively. Volcanic plots, a Venn diagram, and a heatmap were used to show the findings (Figures 1C, D, H, Figures 2C, D; Supplementary Table S4). A total of 883 DE lncRNA, including 334 upregulated and 549 downregulated lncRNA, and 916 DE circRNA, including 317 upregulated and 599 downregulated circRNA, were found in the GSE153761 datasets (Figures 1E, F, Figures 2E, F).

### Construct lncRNA-miRNA-mRNA and circRNA-miRNA-mRNA networks

**FIGURE 3:**
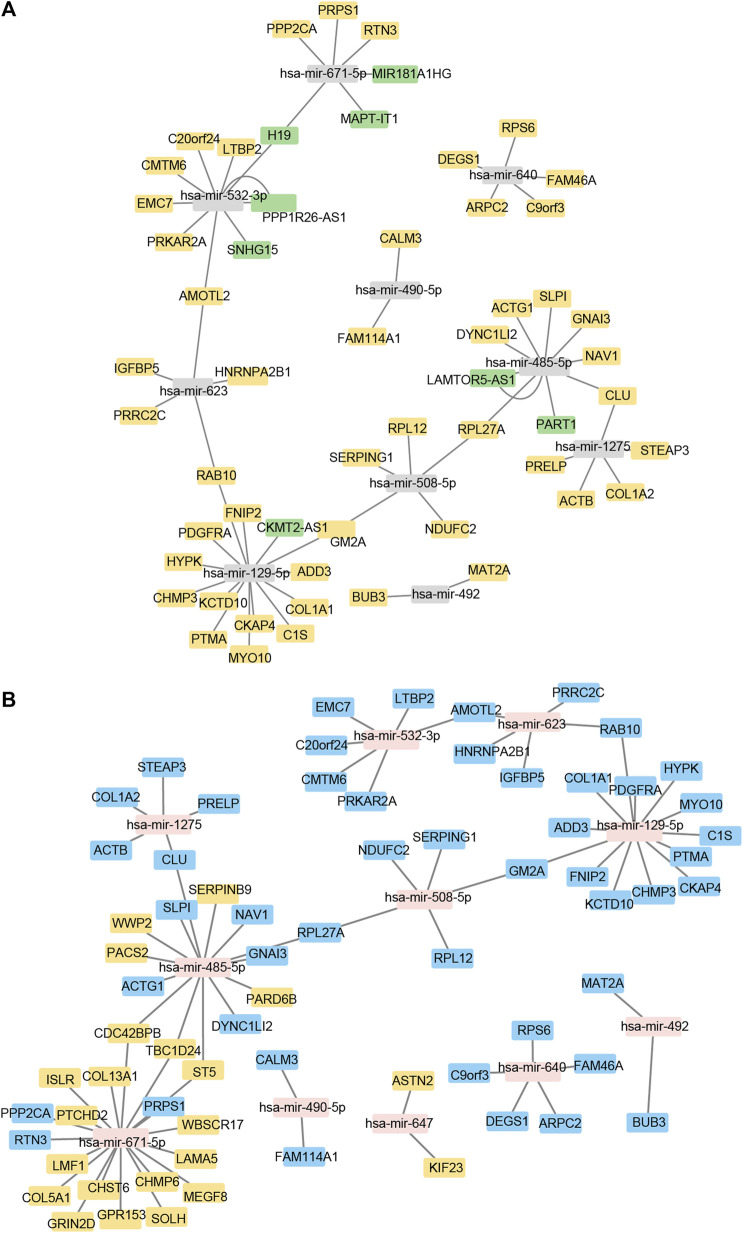
Construction of the lncRNA/cirRNA-miRNA–mRNA regulatory network. **(A)**: CeRNA interaction network of lncRNA-miRNA-mRNA. The mRNA is represented by orange, the miRNA by gray, and the lncRNA by green. **(B)**: mRNA-miRNA-circRNA ceRNA interaction network. mRNA is represented by blue, miRNA by pink, and circRNA by orange.

Using Cytoscape software, a ceRNA network was established based on interactions between miRNA and lncRNA, mRNA and miRNA, and lncRNA and mRNA (Figures 3A, B). There are 8 lncRNA nodes, 48 mRNA nodes, 11 miRNA nodes, 22 cirRNA nodes, and 139 mRNA nodes in the lncRNA/cirRNA-miRNA-mRNA network (Supplementary Tables S5–S7).

### PPI network analysis, GO, and KEGG enrichment analysis of hub genes

**FIGURE 4:**
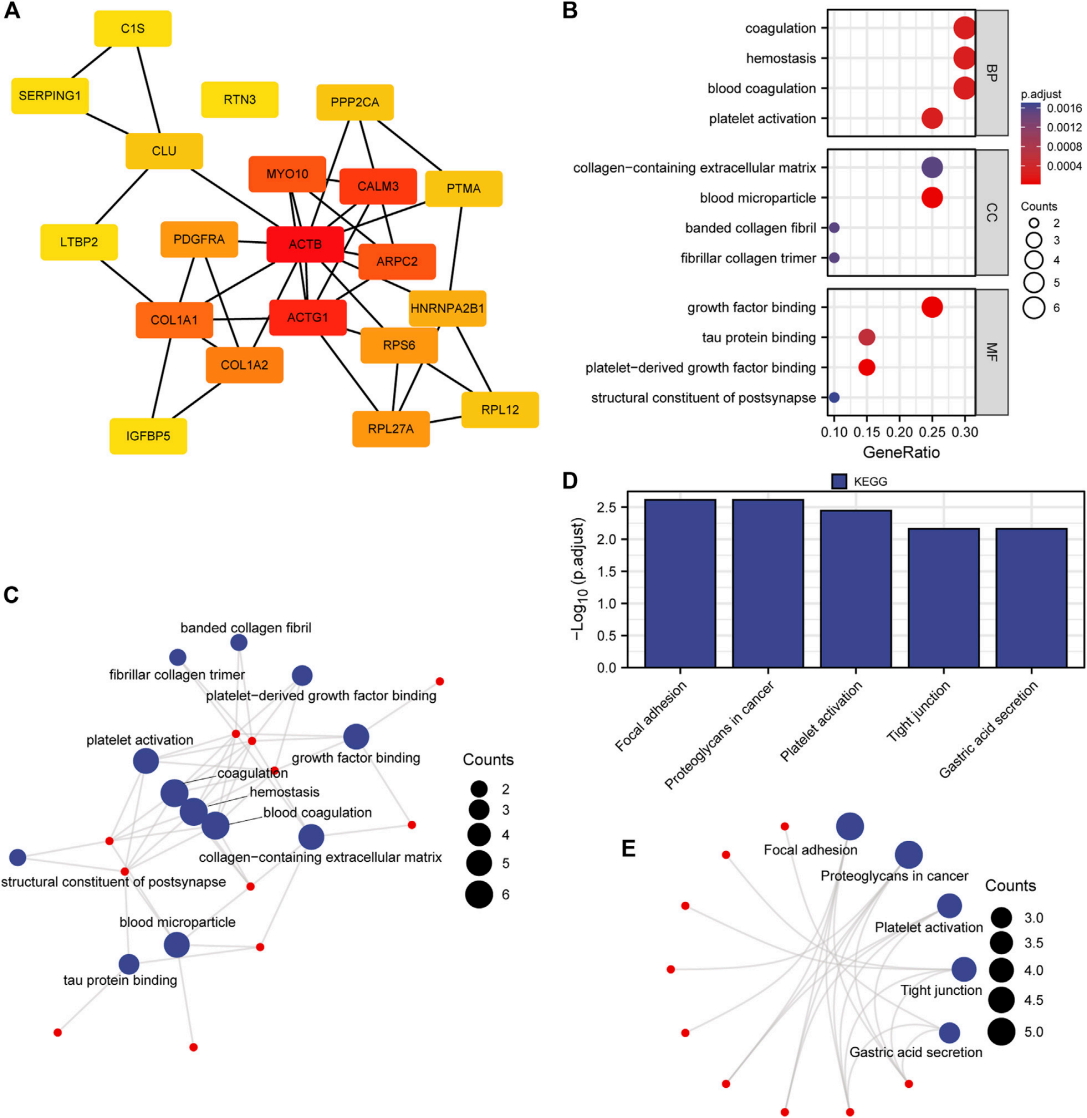
PPI network, GO and KEGG enrichment analysis. **(A)** Nodes with red to yellow colors indicate genes with high to low PPI degree scores. **(B, C)** Hub gene GO enrichment analysis findings were shown in bubble and network diagrams. **(D, E)** Analysis of the 20 hub genes’ enriched KEGG pathways. BP represents a biological process, MF represents a molecular function and CC represents a cellular component.

Considering the importance of hub genes in a network, we utilized an MCODE technique to screen the top 20 hub genes (*ACTB*, *ACTG1*, *CALM3*, *MYO10*, *ARPC2*, *COL1A1*, *COL1A2*, *RPS6*, *PDGFRA*, *RPL27A*, *HNRNPA2B1*, *CLU*, *RPL12*, *PTMA*, *PPP2CA*, *C1S*, *SERPING1*, *RTN3*, *LTBP2*, *IGFBP5*) from the PPI network and Cytoscape software to show the interaction network of hub genes (Figure 4A; Supplementary Table S8).

The findings demonstrated that these hub genes are primarily enriched in BP related to blood coagulation, hemostasis, and coagulation, CC related to blood microparticles, collagen-containing extracellular matrix, and MF related to factor binding, tau protein binding, and structural components of the cytoskeleton (Figures 4B, C; Supplementary Table S9). Additionally, these hub genes were also enriched in KEGG pathways for focal adhesion, cancer-related proteoglycans, and the PI3K-Akt signaling pathway (Figures 4D, E; Supplementary Table S10).

**FIGURE 5:**
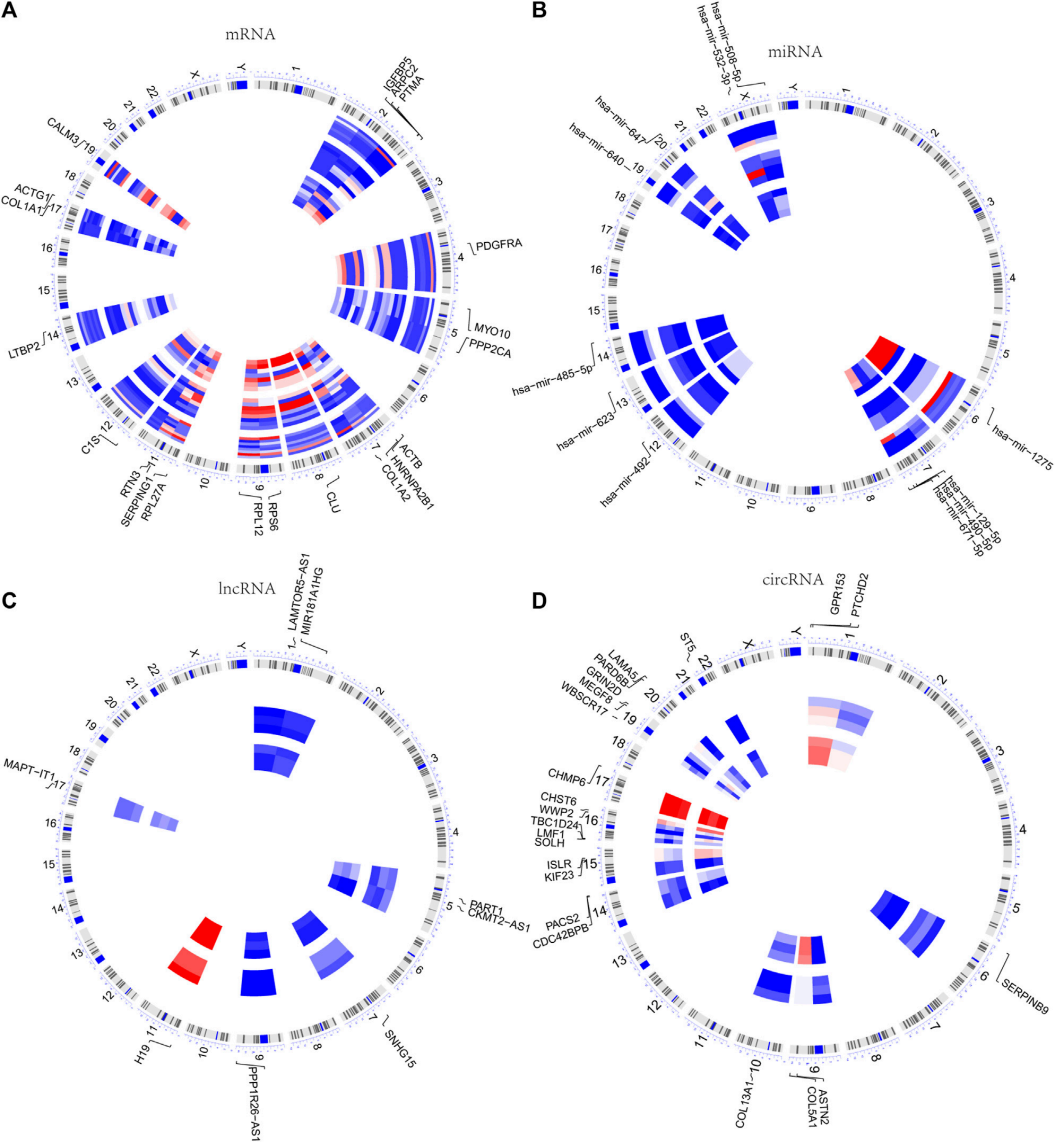
Chromosomal locations of DEGs. **(A)** Chromosomal localization of 20 hub genes in the PPI network. **(B–D)** Chromosomal localization of DE miRNAs, DE lncRNAs, and DE circRNAs in the ceRNA network.

The chromosomal locations of the 20 hub genes as well as DE miRNAs, DE lncRNAs, and DE circRNAs in the ceRNA network were visualized. The findings revealed that the 20 hub genes were mostly distributed in chr2, chr5, and chr7, chr9, chr11, and chr17 (Figure 5A), the DE miRNAs in chr7 and chrX (Figure 5B), the DE lncRNAs in chr1, chr5 (Figure 5C), and the DE circRNAs in chr1, chr9, chr16, and chr19 (Figure 5D).

### GSEA and GSVA

**FIGURE 6:**
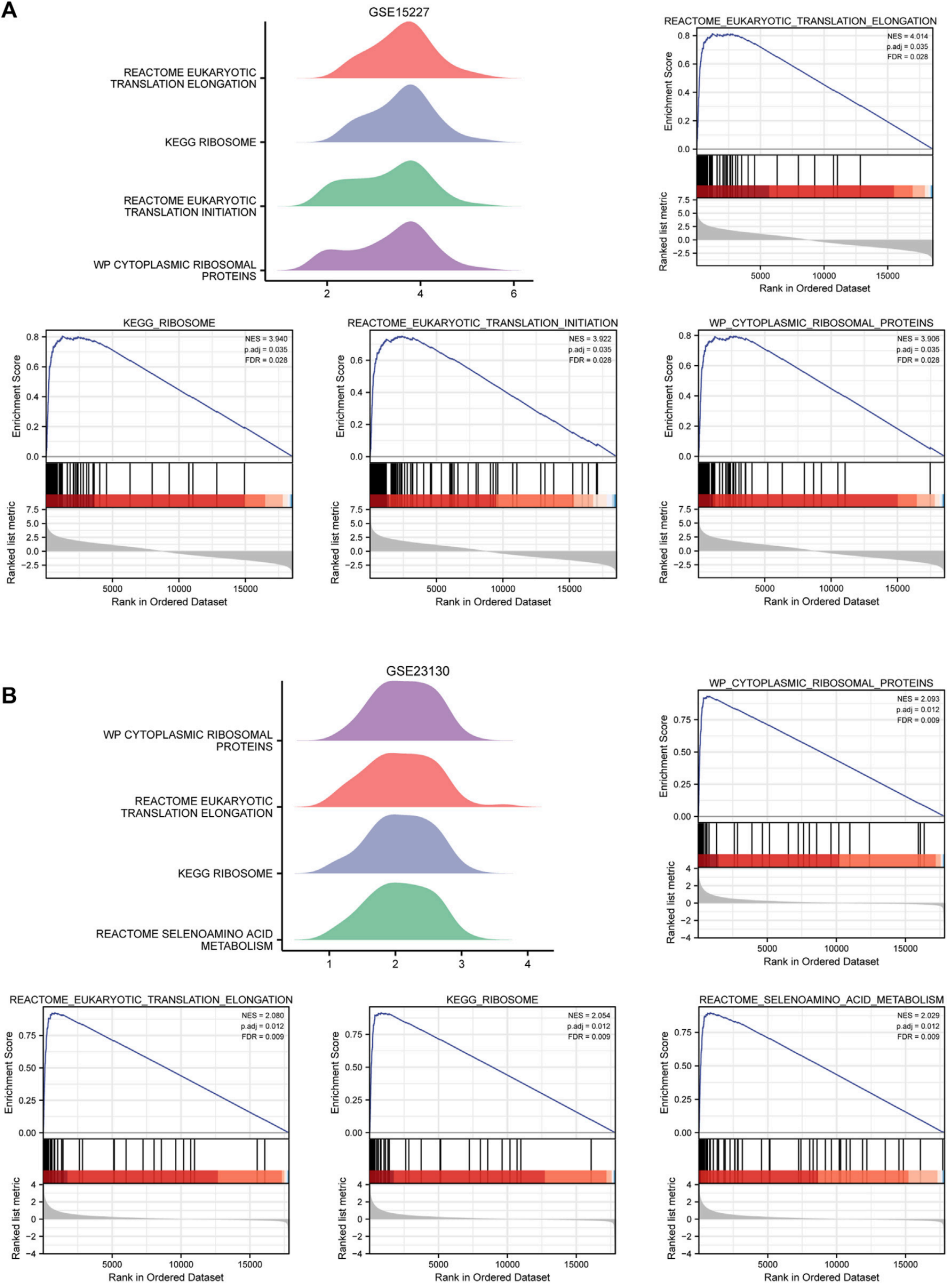
GSEA enriches associated biological processes. **(A)** Four main biological processes—eukaryotic translation elongation, ribosome, eukaryotic translation initiation, and cytoplasmic ribosomal protein—were enriched in the hub genes in GSE15227. **(B)** The hub genes in GSE15227 were particularly enriched for four biological processes, including cytoplasmic ribosomal protein, eukaryotic translation elongation, ribosome, and selenoamino acids metabolism.

GSEA was used to identify the BP and pathways that were significantly different between IDD samples and healthy samples in GSE15227 and GSE23130. The results indicated that the hub genes in GSE15227 were considerably enriched in eukaryotic translation elongation, ribosome, eukaryotic translation initiation, cytoplasmic ribosomal protein (Figure 6A; Supplementary Table S11). Hub genes in GSE23130 were highly enriched in cytoplasmic ribosomal protein, eukaryotic translation elongation, ribosome, and selenoamino acid metabolism (Figure 6B; Supplementary Table S12).

**FIGURE 7:**
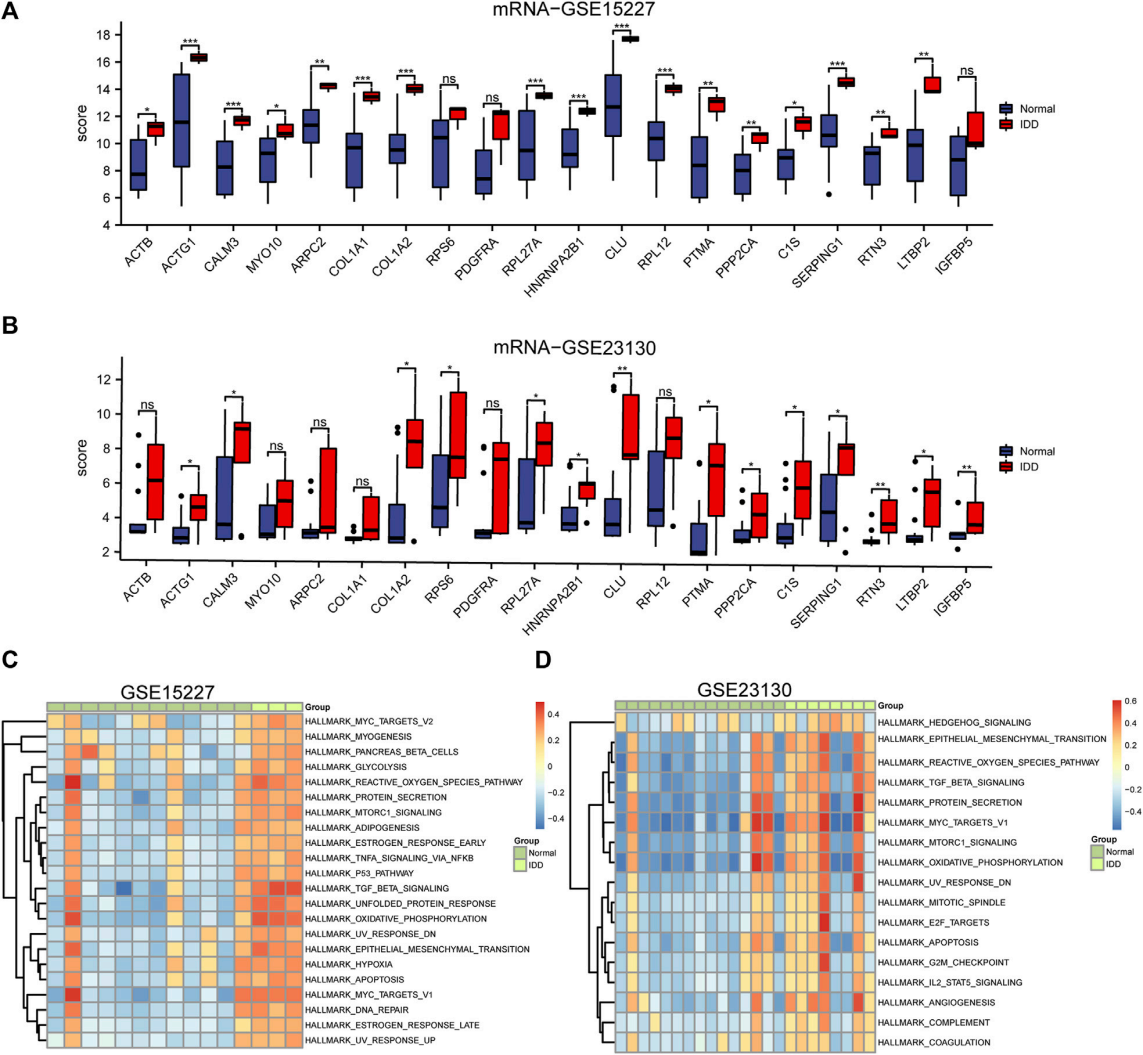
Differential expression analysis of the top 20 hub genes and GSVA analysis. **(A, B)** Boxplots of chosen DEGs from the GSE15227 and GSE23130, where red denotes IDD and blue, normal. * *p* < 0.05, ** *p* < 0.01, *** *p* < 0.001. **(C, D)** 22 hallmark gene sets are indicated by the GSVA of GSE15227, while 17 hallmark gene sets are indicated by the GSVA of GSE23130.

The GSE15227 and GSE23130 datasets were used to investigate the possible role of hub genes in IDD. A total of 12 hub genes were found to have consistent differential expression in both GSE15227 and GSE23130, including *ACTG1*, *CALM3*, *COL1A2*, *RPL27A*, *HNRNPA2B1*, *CLU*, *PTMA*, *PPP2CA*, *C1S*, *SERPING1*, *RTN3*, and *LTBP2* (Figures 7A, B).

We performed GSVA on IDD samples and compared them to healthy control in GSE15227 and GSE23130 database and the results were displayed as a heatmap (Figures 7C, D). Using the median GSVA score, all IDD samples were divided into low and high score groups and 9 hallmark gene sets scored highly in both datasets. Apoptosis, MTORC1 signaling, the reactive oxygen species pathway, oxidative phosphorylation, and TGF-β signaling were among the gene sets with high scores.

### Immune cell infiltration estimation

Immune infiltration analysis using GSE15227 and GSE23130 was carried out to ascertain the differences in immune cell infiltration patterns between patients with IDD and healthy individuals. The ssGSEA approach was used to assess immune infiltration and to determine the enrichment levels of immune cells and immune-related pathways.

**FIGURE 8:**
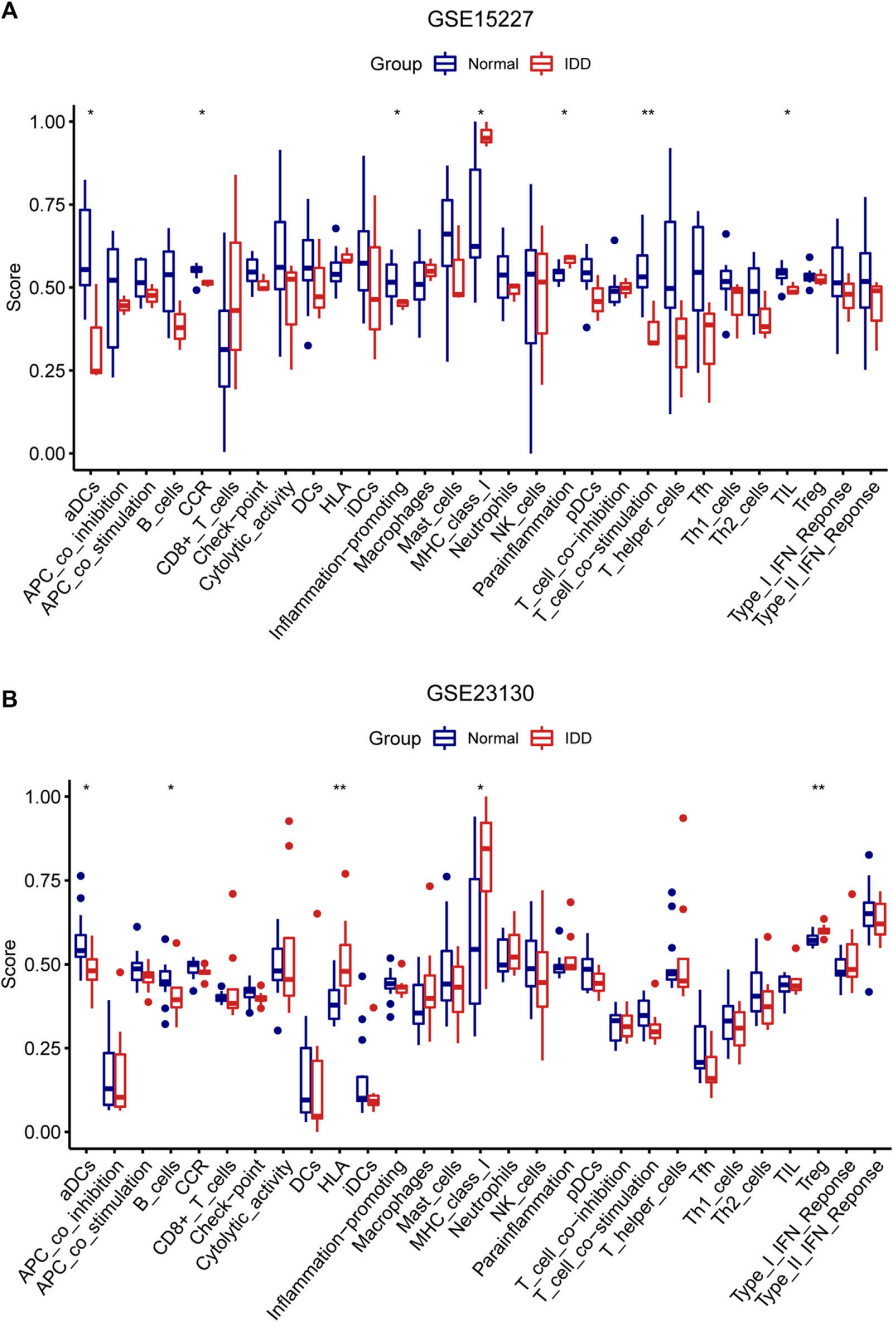
**(A, B)** Analysis of the immune microenvironment in the IDD and normal groups. Comparing the enrichment scores of 16 different immune cell types and 13 immune-related pathways between the two groups in GSE15227 and GSE23130.

The study revealed that immune infiltration, particularly that of activated dendritic cells (aDCs), Th2 cells, and tumor-infiltrating lymphocytes (TILs), was typically lower in the IDD group in GSE15227. Additionally, the IDD group had lower scores for CCR, inflammation-promoting factors, and T-cell co-stimulatory factors, but MHC class I and parainflammation scores revealed the reverse pattern (*p* < 0.05) (Figure 8A). In GSE23130, aDCs and B cells showed reduced levels of immune infiltration, but Treg cells had increased levels. HLA and MHC class I scores were higher in the IDD group (*p* < 0.05) (Figure 8B). It is worth noting that the aDCs had a lower level of immune infiltration both in two of the data sets.

### Construction of mRNA-RBP and mRNA-TF interaction and identification of the potential drugs

**FIGURE 9:**
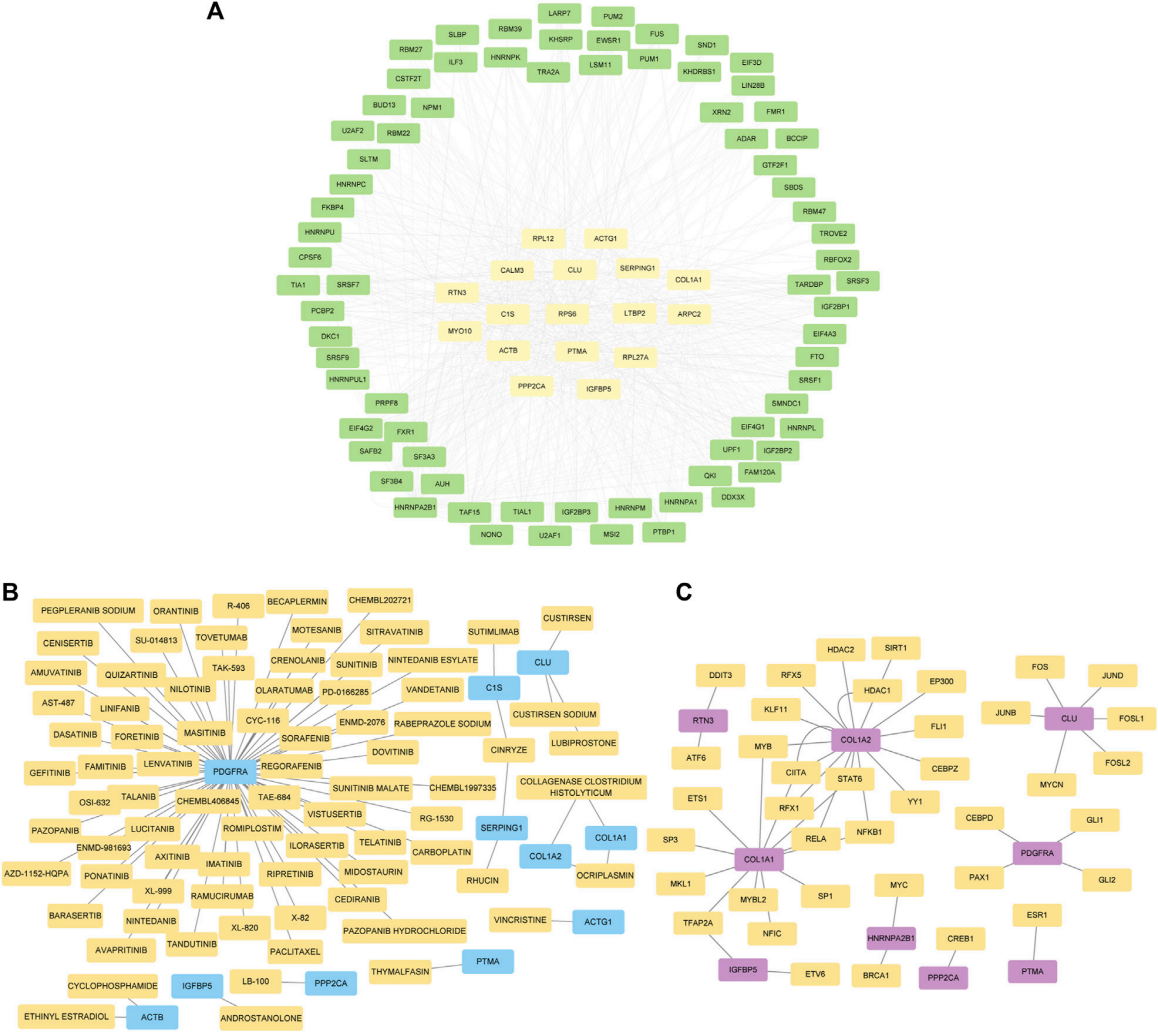
Construction of the mRNA-RBP and mRNA-TF interaction networks and identification of the potential drugs. **(A)** The interaction network between hub genes and RBP, the hub genes are shown in orange, while RBP is represented by green. **(B)** Hub gene potential drug network; the blue represents the hub genes, and the orange represents the potential drug. **(C)** The interaction network between hub genes and TF, purple represents hub genes, orange represents the TF.

Starbase v2.0 database was used to estimate the network of interactions between hub genes and RBP, and Cytoscape was used for visualization. The link between hub genes and RBP were illustrated in mRNA-RBP interaction network (Figure 9A). The DGIdb database shows the relationship between hub genes and known or potential drugs. We discovered 80 possible drugs or chemical compounds that correlate to 11 mRNAs. Among these, two drugs or chemical compounds, such as collagenase *clostridium* histolyticum and ocriplasmin, simultaneously targeted *COL1A1* and *COL1A2*. We also discovered that 66 drugs or chemical compounds specifically targeted the *PDGFRA* (Figure 9B; Supplementary Table S13). Moreover, we investigated potential transcription factor targets for hub genes and the TRRUST database was used to identify a total of 47 connections between transcription factors and hub genes (Figure 9C; Supplementary Table S14).

### RF and ROC curve analysis

**FIGURE 10:**
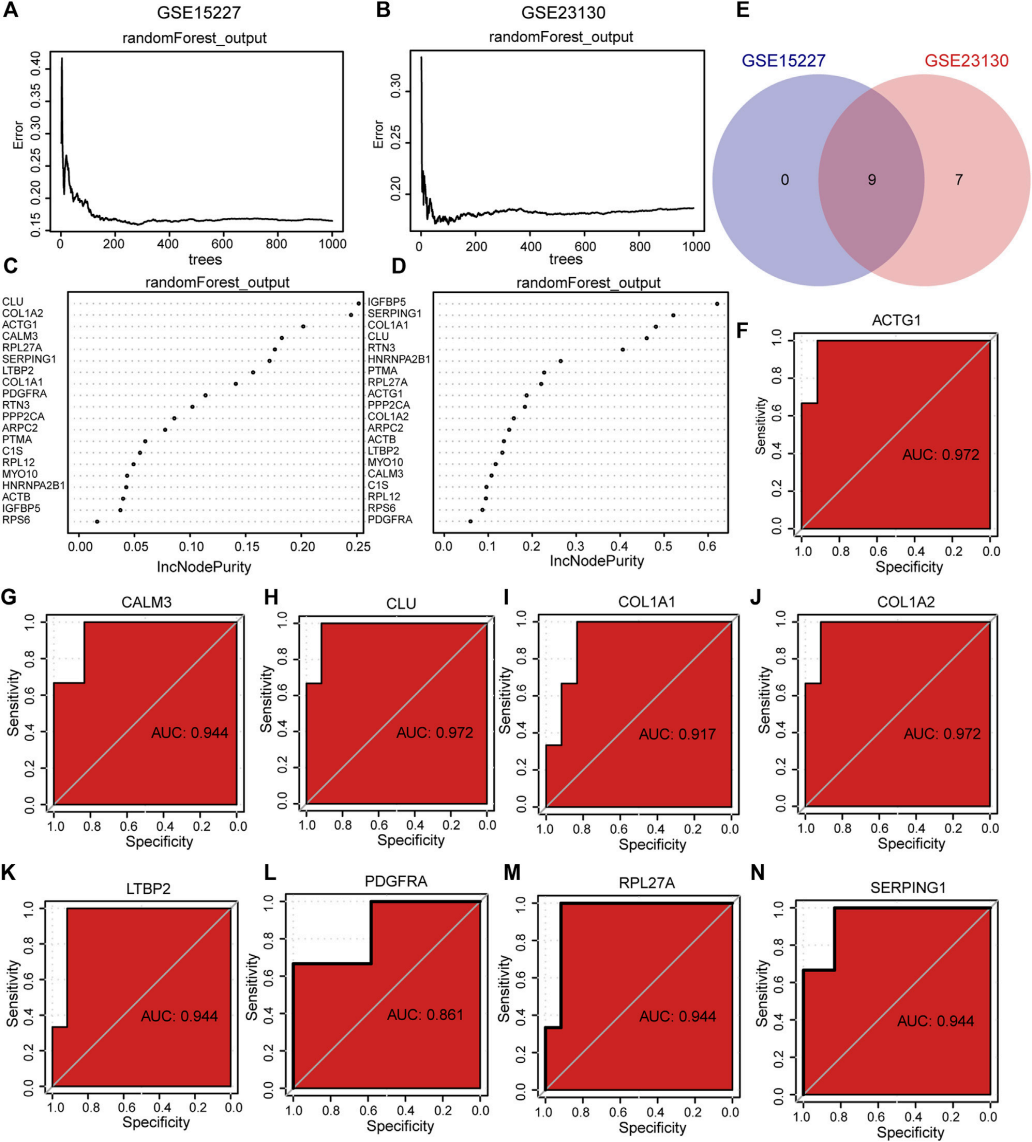
The RF and ROC analysis. **(A, B)** Training error of RF in GSE15227 dataset and GSE23130 dataset. **(C, D)** RF models in GSE15227 and GSE23130 datasets showed hub genes. **(E)** Venn diagram of hub genes obtained by RF analysis. **(F–N)** The ROC curve of the hub genes in the GSE23130 dataset, the closer the AUC in the ROC curve was to 1, the better the diagnostic effect was. RF represents the random forest, ROC represents receiver operating characteristic, AUC represents the area under the curve.

To determine the diagnostic value of hub genes in IDD, we used RF to analyze the expression of 20 hub genes in the GSE15227 and GSE23130 datasets. The results showed that 9 diagnostic markers were obtained in GSE15227 dataset (Figures 10A, B) and 16 diagnostic markers were obtained in GSE23130 datasets (Figures 10C, D). We obtained a total of 9 common hub genes by taking the intersection of the hub genes identified through RF analysis of the GSE15227 and GSE23130 datasets, including ACTG1, CALM3, CLU, C OL1A1, COL1A2, LTBP2, PPP2CA, RPL27A and SERPING1.

**FIGURE 11:**
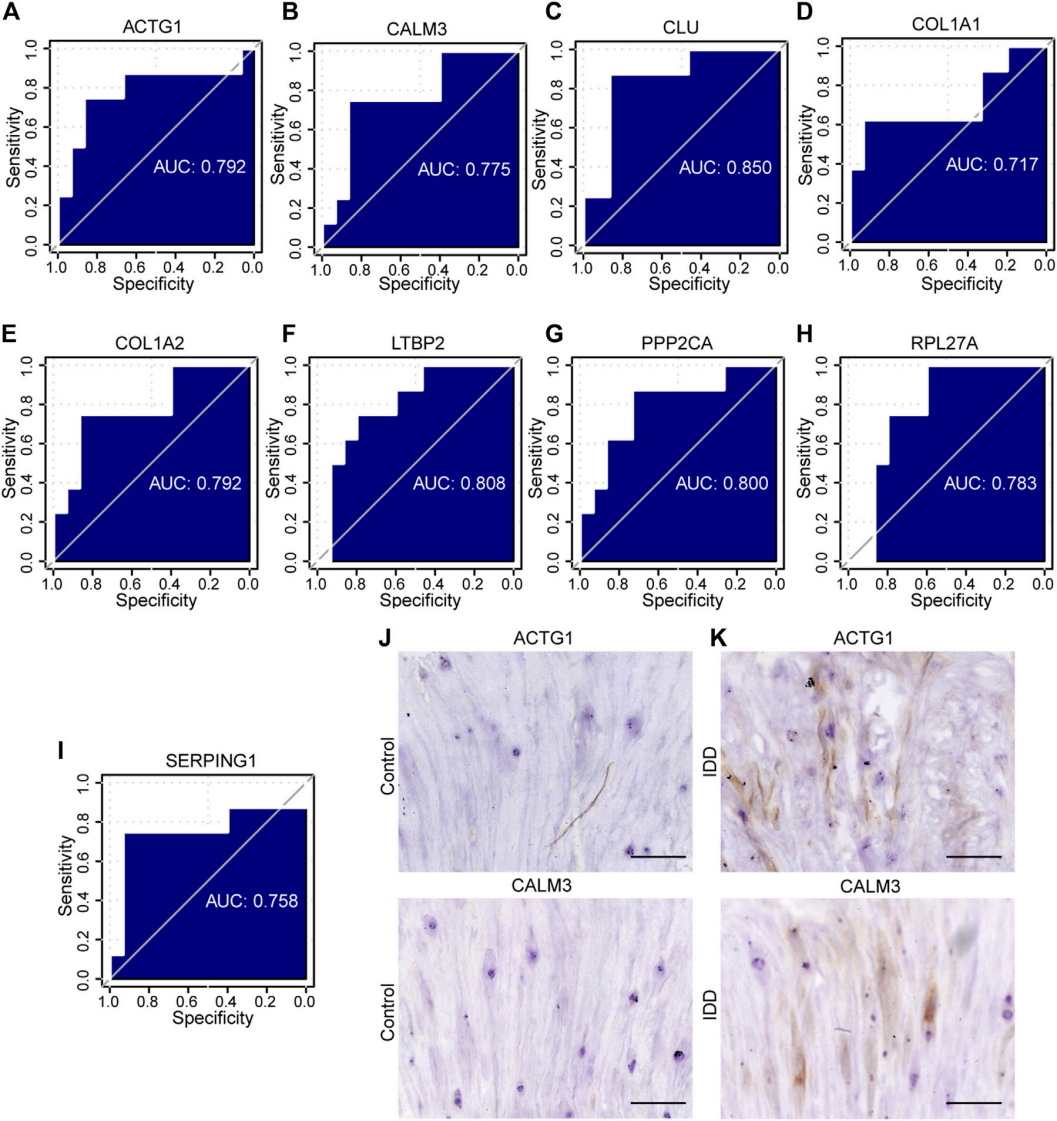
The ROC curve analysis and immunohistochemistry. **(A–I)** The ROC curve of the hub genes in the GSE23130 dataset, the closer the AUC in the ROC curve was to 1, the better the diagnostic effect was. **(J–K)** The immunohistochemical (IHC) staining of ACTG1 and CALM3 both in IDD group and controls, bar = 100 μm. n = 3, ∗ *p* < 0.05, ∗∗*p* < 0.01, ∗∗∗*p* < 0.001. ROC represents receiver operating characteristic, AUC represents the area under the curve.

To assess the diagnostic potential of the expression differences of the identified hub genes in IDD, we further plotted the ROC curves for the 9 hub genes in different groups of GSE15227 and GSE23130 datasets. In the GSE15227 dataset, the expression of ACTG1, CALM3, CLU, and COL1A2 showed high accuracy in diagnosing IDD (Figures 10F–N). On the other hand, in the GSE23130 dataset, the expression of ACTG1, CALM3, and COL1A1 showed high accuracy in diagnosing IDD (Figures 11A–I).

### Validation of the hub genes

**FIGURE 12:**
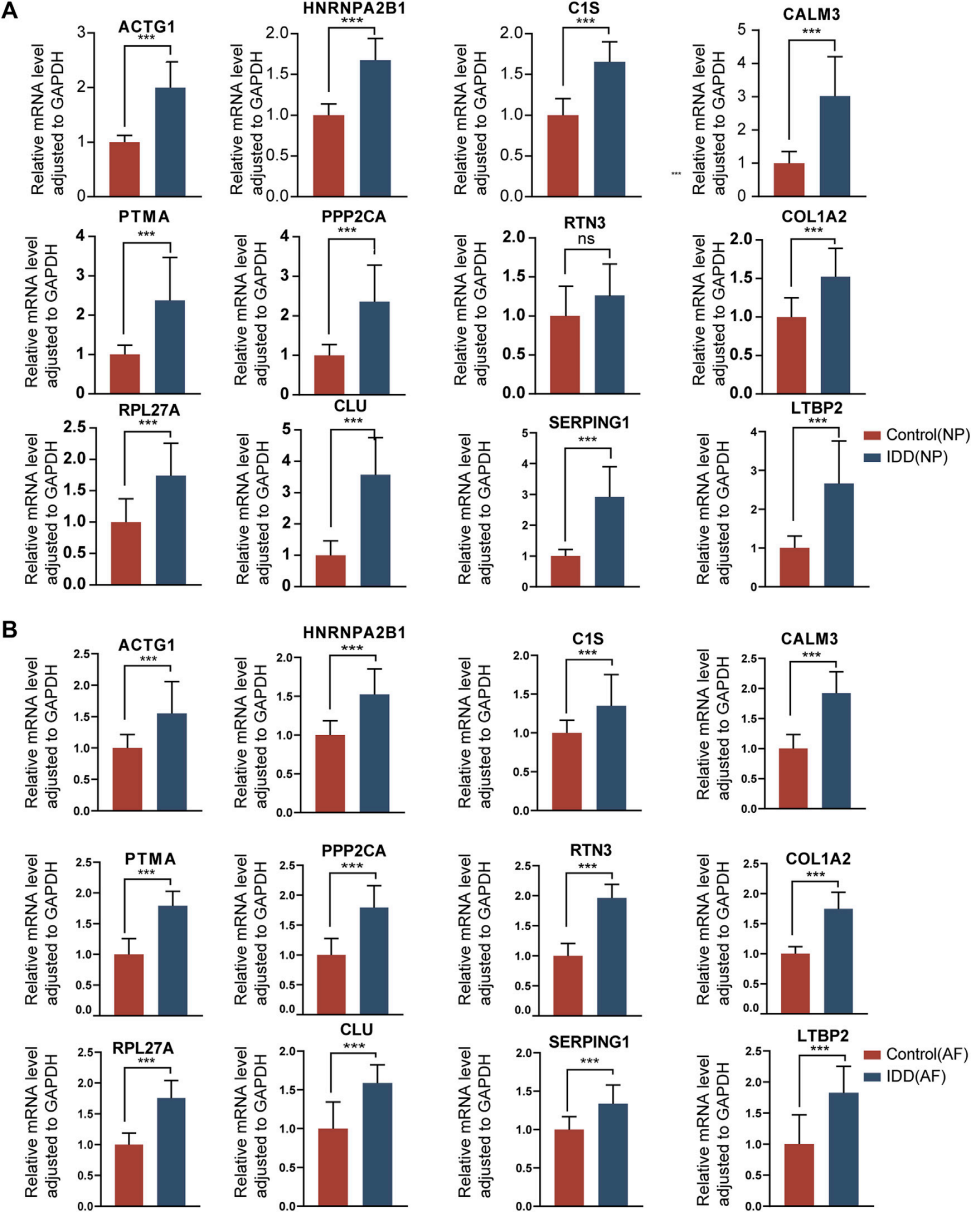
The gene expression of hub genes in normal intervertebral disc tissues and IDD tissues. **(A)**: Expression levels of mRNAs were compared between the two groups in NP tissues. **(B)**: Expression levels of mRNAs were compared between the two groups in AF tissues. ∗ *p* < 0.05, ∗∗*p* < 0.01, ∗∗∗*p* < 0.001.

To validate the identified hub genes, we obtained the RNA from 36 human intervertebral disc tissues, including 12 from patients with Pfirrmann levels I or II-disc degeneration and 24 from patients with levels III or V disc degeneration. The mRNA levels of *ACTG1*, *CALM3*, *COL1A2*, *RPL27A*, *HNRNPA2B1*, *CLU*, *PTMA*, *PPP2CA*, *C1S*, *SERPING1* and *LTBP2* were higher in IDD groups than in healthy control in both NP tissues and AF tissues. In AF tissues, the RTN3 was higher than in the IDD group, while there was no significant difference in NP tissues (Figures 12A, B). To verify the results of RF and ROC curve analysis, we performed IHC staining to detect the expression levels of ACTG1 and CALM3 in IVD tissues. It showed that both the expression levels of ACTG1 and CALM3 were increased in IDD group than controls (Figures 11J, K).

## Discussion

IDD is a significant contributor to low back pain, which has considerable social and financial costs (Cohen, 2022). With a growing frequency in the aging population, there is an urgent need to determine the etiology and optimal treatment for IDD. Unfortunately, the diagnosis of IDD is mostly based on symptoms and imaging, which makes early detection and therapy difficult (Lan et al., 2022). Although multiple research using human participants concluded that macrophages are the most critical players in IDD, the role of other immune cells in the development of IDD remains unknown (Silva et al., 2019; Wang et al., 2021). In this study, we acquired IDD hub genes, build a ceRNA network, and study immune infiltration and potential drugs using bioinformatics analysis. These results revealed that hub genes such as *ACTG1*, *CALM3*, *COL1A2*, *RPL27A* and others were associated with IDD and immune cells including Treg cells, dendritic cells, Th2 cells and tumour-infiltrating lymphocytes are involved in the process of IDD. In addition, the PDGFRA and two potential drugs were found to be significant in IDD development.

Non-coding RNA is essential in the process of different diseases (Huang et al., 2021; Zhong et al., 2022). The involvement of lncRNA/circRNA-miRNA-mRNA regulatory networks and predicted multiple ceRNA regulatory axes was revealed to be substantial in NP cells isolated from IDD patients (Huo et al., 2021). The lnc RNA PART1 has been shown to influence NP cell degeneration through the miR-93/MMP2 pathway (Gao et al., 2020). It was discovered that the *lncRNA SLC20A1* targets the *miR-31-5p/MMP3* axis to enhance extracellular matrix breakdown in NP cells (Yang et al., 2019). Also, a study revealed that *LINC00969* accelerates intervertebral disc degeneration via sponging *miR335-3p* and controlling *NLRP3* inflammasome activation (Yu et al., 2019). Apart from these studies on lncRNA, many others have shown that circRNAs act as miRNA sponges to influence the pathophysiology of IDD. For example, *circRNA 104670* could directly bind to *miR-17-3p* and reverse the negative regulation of *miR-17-3p* on *MMP-2*, thus inhibiting the apoptosis of NP cells (Song et al., 2018). Bioinformatics investigation revealed that matrix metalloproteinase 2 (MMP2) was a possible target of miR185p, and metallopeptidases 2 (circTIMP2) were shown to increase TNF-α- and IL-1β-induced NP cell imbalance between ECM anabolism and catabolism through miR-185p-MMP2 signaling (Guo W. et al., 2020). Nonetheless, there is still a lack of thorough knowledge of the molecular interactions involved in IDD since additional key functional regulatory axes may exist and a disease-wide regulatory network is required. Our research analyzed the genes and non-coding RNAs, then processed the data from public sources to form a molecular interaction network. The network included 48 DE mRNA, 11 DE miRNA, 8 DE lncRNA, 22 DE circRNA, and 142 potential lncRNA/circRNA-miRNA-mRNA axes. We are excited to use this network to identify the genes and ncRNAs that are most likely connected to IDD, laying the groundwork for future studies.

While *ACTG1* is a non-muscle actin gene, it is a member of the actin family. *ACTG1* has been shown to have a variety of roles recently. Loss of hearing is connected to the *ACTG1* mutation (Zhu et al., 2003). *ACTG1* knockdown prevents tumor cells from migrating, proliferating, and repairing wounds via the ROCK signaling system (Dong et al., 2018). In this study, both the level of *ACTG1* and PI3K-Akt signaling pathway were higher in the IDD group. Bioinformatics analysis revealed ACTG1 highly associated with the PI3K-Akt signaling pathway (Parrini et al., 2016; Hou et al., 2022). It is speculated that ACTG1 may play a role in the development of IDD by controlling the PI3K/AKT signaling pathway, and further research is needed to be demonstrated *in vivo* and *in vitro* experiments. Moreover, it has been shown that the development and progression of IDD are highly related to oxidative stress (ROS). Excessive ROS play a key role as mediators in the cell signaling network (Zhong et al., 2023). They control the senescence, apoptosis, autophagy, and proinflammatory phenotypes of disc cells (Feng et al., 2017; Liu Y. et al., 2019; Dai et al., 2021). According to the report, *HNRNPA2B1*, a nuclear reader and effector of the m6A mark, may have a significant role in controlling mitochondrial stress and endothelial cell permeability as well as enhancing the secretion of inflammatory cytokines (Alarcón et al., 2015). *HNRNPA2B1* was identified in this work by bioinformatics analysis, and it was hypothesized that this hub gene is connected to mitochondrial dysfunction and inflammatory response-mediated IDD pathogenesis. Also, our research has shown that the expression of various coding genes, including *CALM3*, *MYO10*, *ARPC2*, *RPS6*, *RPL27A*, and *PDGFRA*, was increased in IDD; however, it is yet unclear how these hub genes contribute to the occurrence of IDD. Further research on these crucial genes and their signaling networks may open up new avenues for the treatment of IDD.

Throughout the course of IDD, several kinds of immune cells, such as neutrophils, T cells, and macrophages, govern the immunological response. Immune cell infiltration may produce a substantial number of proinflammatory chemicals, promoting an inflammation cascade inside the disc (Hai et al., 2022). A significant association between macrophages and progenitor NP cells was discovered through NF-B signaling pathways during the development of IDD (Ling et al., 2021). The interaction between macrophages and IVD polarized macrophages toward a more proinflammatory state, which accelerated IVD degradation. In this research, there was a significant amount of Treg cell infiltration in the IDD group, but there was no difference in macrophage infiltration in the intervertebral disc between the two groups. Similarly to our findings, several academics have linked the Tregs to the development of IDD (Wang et al., 2021). Moreover, several other immune cell types, such as neutrophils and T cells, have drawn a lot of interest (Duan et al., 2022). Eventually, it is anticipated that the development of immune cell responses will lead to a breakthrough in the treatment of IDD.

When disc tissue is exposed to extreme mechanical strain, mitochondrial fusion occurs. Protein downregulation causes mitochondrial fusion abnormalities, which cause NP cell damage. Mitochondria-related genes, such as SOX9, FLVCR1, NR5A1, and UCHL1, play an important role in the progress of IDD (Zhu et al., 2022). There was a strong positive correlation between MFN2 and the level of immune infiltration of three types of invasive immune cells and the function of regulating mitochondrial fusion (Guo et al., 2023). In our study, we found that *COL1A2*, *HNRNPA2B1*, *CLU*, *PPP2CA*, and *RTN3.* Hub genes including COL1A2, HNRNPA2B1, CLU, PPP2CA, and RTN3 have been linked to mitochondrial dysfunction in a variety of disorders (Sharoar et al., 2016; Garros et al., 2017; Ren et al., 2019; Wang et al., 2020; Moriggi et al., 2021). Future research will focus on how these genes contribute to mitochondrial dysfunction-induced IDD.

The top hub gene for these molecules or pharmaceuticals is PDGFRA, which is under the control of 66 potential drugs or molecular compounds. This gene encodes a tyrosine kinase receptor on the cell surface for members of the platelet-derived growth factor family and is involved in organ development, wound healing, and tumor growth (Ko et al., 2020). *PDGFRA* interacts with PI3K/AKT and STAT proteins to regulate cell migration, proliferation, and survival (Zeng et al., 2019). Moreover, as it has been shown that members of the PI3K family play a role in the development of IDD, it is presumed that *PDGFRA* played a role in the development of IDD by controlling the PI3K/AKT signaling pathways. Using the DGIdb online database, we performed a drug-gene interaction study and discovered two related drugs, collagenase *clostridium* histolyticum and ocriplasmin, for the treatment of IDD. Ocriplasmin is mostly used to treat various ophthalmic illnesses (Pirani et al., 2019), while collagenase *clostridium* histolyticum is primarily used to treat Dupuytren’s contracture or Peyronie’s disease (Abdel Raheem et al., 2018; Fletcher et al., 2019). Collagenase *clostridium* histolyticum and ocriplasmin have reportedly been identified as possible treatments for osteoarthritis (OA) (Xu et al., 2022). Nevertheless, further research is required to determine if these drugs have sufficient effects on IDD.

Compared to previous studies, we conducted a comprehensive exploration of various types of RNA, including not only mRNA analysis but also miRNA, lncRNA, and circRNA (Yang et al., 2023). By integrating multiple RNA types, we can gain a more comprehensive understanding of the molecular mechanisms involved in IDD. This approach allows for a broader exploration of gene features and potential regulatory networks associated with IDD, which may provide a unique contribution compared to studies focusing solely on mRNA (Li et al., 2019). Additionally, we constructed lncRNA/circRNA-miRNA-mRNA regulatory networks and PPI networks. By integrating various resources and databases, we can gain a more comprehensive understanding of potential interactions and regulatory pathways related to IDD (Zhan et al., 2021). This integrative approach enhances the reliability and robustness of the research findings. We also utilized the DGidb database to predict potential drugs or small molecule compounds that interact with hub genes. IDD is a complex disease, and individual patients may respond differently to drugs. By analyzing the genetic profiles of patients, we can determine which drugs are more suitable for specific individuals. Furthermore, studying the interaction between drugs and genes can lead to the discovery of new drug targets and the development of treatment strategies tailored to specific genetic variations (Cotto et al., 2018). Finally, we performed experimental validation using RT-qPCR and immunohistochemistry. This experimental validation enhances the reliability and significance of the bioinformatics results, providing valuable confirmation to our discoveries.

This research has several drawbacks. This research was entirely reliant on public datasets with a limited sample size, which might contribute to high false-positive rate and one-sided results, thus, it is necessary to improve detection power by integrating multiple individual databases in a future study. Second, further experiments are needed to verify the role of biomarkers, such as ACTG1, CALM3, CLU, in IDD. Finally, our research did not take into account several factors, including sex, age, and the underlying condition of IDD and we will take these factors into account in the future.

## Conclusion

In conclusion, the current work has investigated the molecular processes underlying IDD using thorough bioinformatics analysis. We screened 20 hub genes associated with IDD and examined their functions and enriched pathways. Based on comprehensive bioinformatics research, the IncRNA/circRNA-miRNA-mRNA network, important regulatory mechanisms, and immune infiltration features of IDD were also discovered. Also, the DGIdb database was used to find two potential drugs, *clostridium* histolyticum and ocriplasmin. All in all, these discoveries might provide a fresh viewpoint for IDD diagnosis and therapy.

## Data Availability

The original contributions presented in the study are included in the article/Supplementary Material, further inquiries can be directed to the corresponding authors.
