# Supplementary material for: Identification of novel gene signatures and immune cell infiltration in intervertebral disc degeneration using bioinformatics analysis
Source: Front Mol Biosci. 2023 Jul 14;10:1169718. doi: 10.3389/fmolb.2023.1169718 (PMC10380950; doi:10.3389/fmolb.2023.1169718)
Supplement: Supplementary file 1 [file Table1.DOCX]

Table S1. Data Information Sheet

|  | Location | Molecule | Control | | IDD | |
| --- | --- | --- | --- | --- | --- | --- |
|  |  |  | Sample Size | Grades | Sample Size | Grades |
| GSE15227 | disc tissue | mRNA | 12 | Ⅱ | 3 | III-Ⅳ |
| GSE23130 | disc tissue | mRNA | 15 | Ⅰ-Ⅱ | 8 | III-Ⅳ |
| GSE19943 | nucleus pulposus | miRNA | 3 | Ⅱ | 3 | Ⅳ |
| GSE116726 | nucleus pulposus | miRNA | 3 | Ⅰ-Ⅱ | 3 | III-Ⅳ |
| GSE153761 | cartilage endplate | IncRNA/circRNA | 3 | Ⅰ-Ⅱ | 3 | III-Ⅳ |

IDD: intervertebral disc degeneration

**Table S2: Primers used for RT-qPCR**

| ACTG1 | F:5′- CACGAGACCACCTTCAACTCCATC -3′ |
| --- | --- |
|  | R:5′- ACTCCTGCTTGCTAATCCACATCTG -3′ |
| CALM3 | F:5′- CGATGAGGAGGTGGATGAGATGAT -3′ |
|  | R:5′- CAGGGAAGAAGGAGAAAGAGCAATC -3′ |
| COL1A2 | F:5′- AGGTGTAAGCGGTGGTGGTTATGA -3′ |
|  | R:5′- TTGGGTCTGAGAGAAGGTGCTGAG -3′ |
| RPL27A | F:5′- AATGGACGGGACTTGGAGACTGAA -3′ |
|  | R:5′-AGCACTGGAATCGGCGGACTTA -3′ |
| HNRNPA2B1 | F:5′- GTTATGGAGGAGGAAGAGGAGGATA -3′ |
|  | R:5′- CCGTAGTTAGAAGGTTGCTGGTT -3′ |
| CLU | F:5′- GCTCTTCCAGGACAGGTTCTTCA -3′ |
|  | R:5′-GGTCATCGTCGCCTTCTCGTAT -3′ |
| PTMA | F:5′- GGTGATGGTGAGGAAGAGGATGGA -3′ |
|  | R:5′- AGACGGGAAGTGGAGGGTGAATAG -3′ |
| PPP2CA | F:5′- TTGCTCTGTCACTCTATGCCCTTT -3′ |
|  | R:5′- TCACAAGTCCCATTCTGCTAACCA -3′ |
| C1S | F:5′-CCTATGGGCTCTACACACGGGTAA -3′ |
|  | R:5′-AGCAAGGCGTCTCAACAATCAAGT -3′ |
| SERPING1 | F:5′- GGACCTGTTACTCTCTGCTCTGACT -3′ |
|  | R:5′- TAAGGAGGCTGGCGATGCTGAA -3′ |
| RTN3 | F:5′- GCAGCCCTCAAATCCTATCTTCCT -3′ |
|  | R:5′- TCCCTCTCAGTCCCACTTTCCAT -3′ |
| LTBP2 | F:5′-AAGTTCTGCCACCTGCCTATCC -3′ |
|  | R:5′-GTCTCCACGCTGTTCTCCACTA-3′ |
| GAPDH | F:5′- ACTTTGGTATCGTGGAAGGACTCA -3′ |
|  | R:5′- CCAGTAGAGGCAGGGATGATGTT -3′ |

**Table S3. Common differential mRNA in GSE15227 dataset and GSE23130 dataset**

| Gene ID | | | | | |
| --- | --- | --- | --- | --- | --- |
| TSPAN2 | SERPING1 | PPT1 | LRP11 | FBXO46 | CHI3L1 |
| TRPS1 | SERPINB1 | PPP6C | LOC100133315 | FBXO28 | CHD4 |
| TRMT112 | SERF2 | PPP3CA | LOC100129292 | FAM98A | CHCHD2 |
| TRIM29 | SEC62 | PPP2CA | LGALS1 | FAM46A | CDK2AP1 |
| TRAM1 | SEC13 | PPIC | LASP1 | FAM127A | CDH11 |
| TPP1 | SCRG1 | PPIA | KTN1 | FAM114A1 | CDC42 |
| TPM3 | SCARA3 | PPARD | KPNB1 | ERH | CD99 |
| TPD52L1 | SAP30L | POLR2G | KMT2C | EPN1 | CD63 |
| TP53 | SAP18 | PMEPA1 | KDELR2 | EPDR1 | CD164 |
| TNPO2 | SAMD4A | PLS3 | KCTD10 | EPB41L2 | CCT5 |
| TMSB10 | SACM1L | PLEKHO2 | KANK1 | EMC7 | CCT2 |
| TMEM47 | S100A4 | PIK3CD-AS1 | ITPK1 | EIF4H | CCND2 |
| TMEM45A | RUNX3 | PHPT1 | ITM2B | EIF4B | CAPZA1 |
| TMEM267 | RUFY1 | PHLDB1 | ITGB5 | EIF2AK1 | CANX |
| TMEM248 | RTN4 | PHF3 | ITGA11 | EIF1 | CAMK1D |
| TMEM245 | RTN3 | PGK1 | IGFBP5 | EEF1A1 | CALM3 |
| TMEM214 | RPS6 | PGAM1 | IFITM3 | DYSF | C9orf3 |
| TMED7 | RPS27L | PFN2 | IFITM2 | DYNLT1 | C6orf48 |
| TMED2 | RPS2 | PDLIM5 | IFITM1 | DYNLRB1 | C20orf24 |
| TMED10 | RPS17 | PDGFRA | IDS | DYNLL1 | C1S |
| TIMP3 | RPS11 | PDGFC | HYPK | DYNC1LI2 | BUB3 |
| TIMP2 | RPN1 | PCBP2 | HTRA1 | DYNC1I2 | BTG1 |
| TIMMDC1 | RPLP0 | PAPSS1 | HPRT1 | DSTN | BNC2 |
| TIMM17A | RPL39L | PAM | HPF1 | DLC1 | BMI1 |
| THY1 | RPL38 | PAK2 | HNRNPK | DIDO1 | B2M |
| THBS2 | RPL35 | NUCKS1 | HNRNPH1 | DIABLO | ATXN10 |
| TGOLN2 | RPL27A | NREP | HNRNPDL | DHX40 | ATP6V0E1 |
| TGFBI | RPL18 | NPTN | HNRNPA2B1 | DEGS1 | ATP6AP1 |
| TGFB1I1 | RPL12 | NORAD | HMGN2 | DDX5 | ATP5J2 |
| TCTN1 | RPL10 | NFIA | HLA-DQA1 | DCN | ATP1B1 |
| TCF4 | RPA2 | NFE2L1 | HLA-B | DAZAP2 | ATP1A1 |
| TCEB2 | RHOBTB3 | NEAT1 | HLA-A | DAXX | ATG12 |
| TBC1D20 | RHEB | NDUFC2 | HIPK3 | DAG1 | ASPN |
| SULF2 | RGS5 | NDUFB8 | HIF1A | CYP1B1 | ASPH |
| SULF1 | REXO2 | NDUFAB1 | H2AFY | CUX1 | ASAP1 |
| STEAP3 | RERE | ND2 | H19 | CUL3 | ARPC2 |
| STAT1 | REEP5 | NCL | GSTP1 | CTSB | ARL6IP5 |
| SSR4 | RCAN1 | NAV1 | GRB2 | CTGF | ARF4 |
| SRPRA | RBPJ | NARS | GPX8 | CSDE1 | APLP2 |
| SRGN | RBM5 | MYO10 | GPX1 | CRTAP | AP3S1 |
| SPG7 | RBBP7 | MYL6B | GPBP1 | COX7A2 | AP2S1 |
| SPARC | RAB5A | MXRA5 | GOLPH3 | COX5A | ANXA4 |
| SOX9 | RAB31 | MSN | GNS | COX1 | ANXA2 |
| SNAPC5 | RAB28 | MIA | GNB1 | COL8A2 | AMOTL2 |
| SMOC2 | RAB10 | MGP | GNAI3 | COL6A3 | AKAP13 |
| SMAD6 | PXYLP1 | MGAT4EP | GM2A | COL5A1 | AFTPH |
| SLPI | PTTG1IP | MCL1 | GLIS2 | COL3A1 | AEBP1 |
| SLC40A1 | PTPRK | MCAM | GLI3 | COL2A1 | ADD3 |
| SLC39A6 | PTPN9 | MATR3 | GJA1 | COL1A2 | ADD1 |
| SLC38A6 | PTMA | MATN2 | GAPDH | COL1A1 | ADAR |
| SLC38A1 | PSMB5 | MAT2B | GABARAP | COL11A1 | ADAMTS5 |
| SH3KBP1 | PSMB4 | MAT2A | FSTL1 | CMTM6 | ACTR3 |
| SH3BGRL3 | PRRC2C | MANF | FOXP1 | CLU | ACTG1 |
| SGK1 | PRPS1 | MAGED1 | FNIP2 | CLTC | ACTB |
| SGCB | PROS1 | MAFB | FNDC1 | CLIC1 | ACSL3 |
| SETD3 | PRNP | MAF | FN1 | CKAP4 | ACKR3 |
| SET | PRKAR2A | LUM | FLRT2 | CHMP3 | ABI3BP |
| SESN2 | PRELP | LTBP2 | FHL1 |  |  |

**Table S4. Common differential miRNA in GSE19943 dataset and GSE116726 dataset**

| Gene ID | | | |
| --- | --- | --- | --- |
| hsa-miR-671-5p | hsa-miR-638 | hsa-miR-508-5p | hsa-miR-485-5p |
| hsa-miR-647 | hsa-miR-623 | hsa-miR-492 | hsa-miR-129-5p |
| hsa-miR-640 | hsa-miR-532-3p | hsa-miR-490-5p | hsa-miR-1275 |

**TableS5. mRNA-miRNA-lncRNA interaction network nodes.**

| mRNA |  | miRNA |  | miRNA |  | lncRNA |
| --- | --- | --- | --- | --- | --- | --- |
| STEAP3 | - | hsa-mir-1275 |  | hsa-mir-671-5p | - | MAPT-IT1 |
| SLPI | - | hsa-mir-485-5p |  | hsa-mir-671-5p | - | MIR181A1HG |
| SERPING1 | - | hsa-mir-508-5p |  | hsa-mir-532-3p | - | PPP1R26-AS1 |
| RTN3 | - | hsa-mir-671-5p |  | hsa-mir-532-3p | - | PPP1R26-AS1 |
| RPS6 | - | hsa-mir-640 |  | hsa-mir-485-5p | - | PART1 |
| RPL27A | - | hsa-mir-485-5p |  | hsa-mir-532-3p | - | H19 |
| RPL27A | - | hsa-mir-508-5p |  | hsa-mir-671-5p | - | H19 |
| RPL12 | - | hsa-mir-508-5p |  | hsa-mir-485-5p | - | LAMTOR5-AS1 |
| RAB10 | - | hsa-mir-129-5p |  | hsa-mir-485-5p | - | LAMTOR5-AS1 |
| RAB10 | - | hsa-mir-623 |  | hsa-mir-532-3p | - | SNHG15 |
| PTMA | - | hsa-mir-129-5p |  | hsa-mir-129-5p | - | CKMT2-AS1 |
| PRRC2C | - | hsa-mir-623 |  |  |  |  |
| PRPS1 | - | hsa-mir-671-5p |  |  |  |  |
| PRKAR2A | - | hsa-mir-532-3p |  |  |  |  |
| PRELP | - | hsa-mir-1275 |  |  |  |  |
| PPP2CA | - | hsa-mir-671-5p |  |  |  |  |
| PDGFRA | - | hsa-mir-129-5p |  |  |  |  |
| NDUFC2 | - | hsa-mir-508-5p |  |  |  |  |
| NAV1 | - | hsa-mir-485-5p |  |  |  |  |
| MYO10 | - | hsa-mir-129-5p |  |  |  |  |
| MAT2A | - | hsa-mir-492 |  |  |  |  |
| LTBP2 | - | hsa-mir-532-3p |  |  |  |  |
| KCTD10 | - | hsa-mir-129-5p |  |  |  |  |
| IGFBP5 | - | hsa-mir-623 |  |  |  |  |
| HYPK | - | hsa-mir-129-5p |  |  |  |  |
| HNRNPA2B1 | - | hsa-mir-623 |  |  |  |  |
| GNAI3 | - | hsa-mir-485-5p |  |  |  |  |
| GM2A | - | hsa-mir-129-5p |  |  |  |  |
| GM2A | - | hsa-mir-508-5p |  |  |  |  |
| FNIP2 | - | hsa-mir-129-5p |  |  |  |  |
| FAM46A | - | hsa-mir-640 |  |  |  |  |
| FAM114A1 | - | hsa-mir-490-5p |  |  |  |  |
| EMC7 | - | hsa-mir-532-3p |  |  |  |  |
| DYNC1LI2 | - | hsa-mir-485-5p |  |  |  |  |
| DEGS1 | - | hsa-mir-640 |  |  |  |  |
| COL1A2 | - | hsa-mir-1275 |  |  |  |  |
| COL1A1 | - | hsa-mir-129-5p |  |  |  |  |
| CMTM6 | - | hsa-mir-532-3p |  |  |  |  |
| CLU | - | hsa-mir-485-5p |  |  |  |  |
| CLU | - | hsa-mir-1275 |  |  |  |  |
| CKAP4 | - | hsa-mir-129-5p |  |  |  |  |
| CHMP3 | - | hsa-mir-129-5p |  |  |  |  |
| CALM3 | - | hsa-mir-490-5p |  |  |  |  |
| C9orf3 | - | hsa-mir-640 |  |  |  |  |
| C20orf24 | - | hsa-mir-532-3p |  |  |  |  |
| C1S | - | hsa-mir-129-5p |  |  |  |  |
| BUB3 | - | hsa-mir-492 |  |  |  |  |
| ARPC2 | - | hsa-mir-640 |  |  |  |  |
| AMOTL2 | - | hsa-mir-623 |  |  |  |  |
| AMOTL2 | - | hsa-mir-532-3p |  |  |  |  |
| ADD3 | - | hsa-mir-129-5p |  |  |  |  |
| ACTG1 | - | hsa-mir-485-5p |  |  |  |  |
| ACTB | - | hsa-mir-1275 |  |  |  |  |

**TableS6. mRNA-miRNA-circRNA interaction network nodes**

| mRNA |  | miRNA |  | miRNA |  | circRNA |
| --- | --- | --- | --- | --- | --- | --- |
| STEAP3 | - | hsa-mir-1275 |  | hsa-mir-647 | - | ASTN2 |
| SLPI | - | hsa-mir-485-5p |  | hsa-mir-671-5p | - | CDC42BPB |
| SERPING1 | - | hsa-mir-508-5p |  | hsa-mir-485-5p | - | CDC42BPB |
| RTN3 | - | hsa-mir-671-5p |  | hsa-mir-671-5p | - | CHMP6 |
| RPS6 | - | hsa-mir-640 |  | hsa-mir-671-5p | - | CHST6 |
| RPL27A | - | hsa-mir-485-5p |  | hsa-mir-671-5p | - | COL13A1 |
| RPL27A | - | hsa-mir-508-5p |  | hsa-mir-671-5p | - | COL5A1 |
| RPL12 | - | hsa-mir-508-5p |  | hsa-mir-671-5p | - | GPR153 |
| RAB10 | - | hsa-mir-129-5p |  | hsa-mir-671-5p | - | GRIN2D |
| RAB10 | - | hsa-mir-623 |  | hsa-mir-671-5p | - | ISLR |
| PTMA | - | hsa-mir-129-5p |  | hsa-mir-647 | - | KIF23 |
| PRRC2C | - | hsa-mir-623 |  | hsa-mir-671-5p | - | LAMA5 |
| PRPS1 | - | hsa-mir-671-5p |  | hsa-mir-671-5p | - | LMF1 |
| PRKAR2A | - | hsa-mir-532-3p |  | hsa-mir-671-5p | - | MEGF8 |
| PRELP | - | hsa-mir-1275 |  | hsa-mir-485-5p | - | PACS2 |
| PPP2CA | - | hsa-mir-671-5p |  | hsa-mir-485-5p | - | PARD6B |
| PDGFRA | - | hsa-mir-129-5p |  | hsa-mir-671-5p | - | PTCHD2 |
| NDUFC2 | - | hsa-mir-508-5p |  | hsa-mir-485-5p | - | SERPINB9 |
| NAV1 | - | hsa-mir-485-5p |  | hsa-mir-671-5p | - | SOLH |
| MYO10 | - | hsa-mir-129-5p |  | hsa-mir-671-5p | - | ST5 |
| MAT2A | - | hsa-mir-492 |  | hsa-mir-485-5p | - | ST5 |
| LTBP2 | - | hsa-mir-532-3p |  | hsa-mir-671-5p | - | TBC1D24 |
| KCTD10 | - | hsa-mir-129-5p |  | hsa-mir-485-5p | - | TBC1D24 |
| IGFBP5 | - | hsa-mir-623 |  | hsa-mir-671-5p | - | WBSCR17 |
| HYPK | - | hsa-mir-129-5p |  | hsa-mir-485-5p | - | WWP2 |
| HNRNPA2B1 | - | hsa-mir-623 |  |  |  |  |
| GNAI3 | - | hsa-mir-485-5p |  |  |  |  |
| GM2A | - | hsa-mir-129-5p |  |  |  |  |
| GM2A | - | hsa-mir-508-5p |  |  |  |  |
| FNIP2 | - | hsa-mir-129-5p |  |  |  |  |
| FAM46A | - | hsa-mir-640 |  |  |  |  |
| FAM114A1 | - | hsa-mir-490-5p |  |  |  |  |
| EMC7 | - | hsa-mir-532-3p |  |  |  |  |
| DYNC1LI2 | - | hsa-mir-485-5p |  |  |  |  |
| DEGS1 | - | hsa-mir-640 |  |  |  |  |
| COL1A2 | - | hsa-mir-1275 |  |  |  |  |
| COL1A1 | - | hsa-mir-129-5p |  |  |  |  |
| CMTM6 | - | hsa-mir-532-3p |  |  |  |  |
| CLU | - | hsa-mir-485-5p |  |  |  |  |
| CLU | - | hsa-mir-1275 |  |  |  |  |
| CKAP4 | - | hsa-mir-129-5p |  |  |  |  |
| CHMP3 | - | hsa-mir-129-5p |  |  |  |  |
| CALM3 | - | hsa-mir-490-5p |  |  |  |  |
| C9orf3 | - | hsa-mir-640 |  |  |  |  |
| C20orf24 | - | hsa-mir-532-3p |  |  |  |  |
| C1S | - | hsa-mir-129-5p |  |  |  |  |
| BUB3 | - | hsa-mir-492 |  |  |  |  |
| ARPC2 | - | hsa-mir-640 |  |  |  |  |
| AMOTL2 | - | hsa-mir-623 |  |  |  |  |
| AMOTL2 | - | hsa-mir-532-3p |  |  |  |  |
| ADD3 | - | hsa-mir-129-5p |  |  |  |  |
| ACTG1 | - | hsa-mir-485-5p |  |  |  |  |
| ACTB | - | hsa-mir-1275 |  |  |  |  |

**Table S7. ceRNA network mRNA node name**

| mRNA node name | | | | | |
| --- | --- | --- | --- | --- | --- |
| STEAP3 | RPL12 | PRELP | MAT2A | GNAI3 | DYNC1LI2 |
| SLPI | RAB10 | PPP2CA | LTBP2 | GM2A | DEGS1 |
| SERPING1 | PTMA | PDGFRA | KCTD10 | FNIP2 | COL1A2 |
| RTN3 | PRRC2C | NDUFC2 | IGFBP5 | FAM46A | COL1A1 |
| RPS6 | PRPS1 | NAV1 | HYPK | FAM114A1 | CMTM6 |
| RPL27A | PRKAR2A | MYO10 | HNRNPA2B1 | EMC7 | CLU |
| BUB3 | ARPC2 | AMOTL2 | ADD3 | ACTG1 | ACTB |
| CKAP4 | CHMP3 | CALM3 | C9orf3 | C20orf24 | C1S |

**Table S8. The results of protein interaction network analysis(PPI).**

| Rank | Name | Score |
| --- | --- | --- |
| 1 | ACTB | 42 |
| 2 | ACTG1 | 30 |
| 3 | CALM3 | 26 |
| 4 | MYO10 | 24 |
| 4 | ARPC2 | 24 |
| 6 | COL1A1 | 13 |
| 7 | COL1A2 | 8 |
| 8 | RPS6 | 6 |
| 8 | PDGFRA | 6 |
| 8 | RPL27A | 6 |
| 11 | HNRNPA2B1 | 5 |
| 12 | CLU | 4 |
| 12 | RPL12 | 4 |
| 12 | PTMA | 4 |
| 12 | PPP2CA | 4 |
| 16 | C1S | 2 |
| 16 | SERPING1 | 2 |
| 16 | RTN3 | 2 |
| 16 | LTBP2 | 2 |
| 16 | IGFBP5 | 2 |

**Table S9. GO enrichment analysis.**

| ONTOLOGY | ID | | | Description | GeneRatio | BgRatio | pvalue | p.adjust | qvalue |
| --- | --- | --- | --- | --- | --- | --- | --- | --- | --- |
| BP | GO:0030168 | | | platelet activation | 5/20 | 153/18670 | 4.86e-07 | 2.59e-04 | 1.74e-04 |
| BP | GO:0007596 | | | blood coagulation | 6/20 | 336/18670 | 1.02e-06 | 2.59e-04 | 1.74e-04 |
| BP | GO:0007599 | | | hemostasis | 6/20 | 341/18670 | 1.11e-06 | 2.59e-04 | 1.74e-04 |
| BP | GO:0050817 | | | coagulation | 6/20 | 342/18670 | 1.13e-06 | 2.59e-04 | 1.74e-04 |
| BP | GO:0000184 | | | nuclear-transcribed mRNA catabolic process， nonsense-mediated decay | 4/20 | 120/18670 | 7.26e-06 | 0.001 | 8.94e-04 |
| CC | GO:0072562 | | | blood microparticle | 5/20 | 147/19717 | 3.05e-07 | 3.47e-05 | 1.76e-05 |
| CC | GO:0062023 | | | collagen-containing extracellular matrix | 5/20 | 406/19717 | 4.34e-05 | 0.002 | 7.74e-04 |
| CC | GO:0005583 | | | fibrillar collagen trimer | 2/20 | 11/19717 | 5.35e-05 | 0.002 | 7.74e-04 |
| CC | GO:0098643 | | | banded collagen fibril | 2/20 | 11/19717 | 5.35e-05 | 0.002 | 7.74e-04 |
| CC | GO:0098644 | | | complex of collagen trimers | 2/20 | 19/19717 | 1.65e-04 | 0.004 | 0.002 |
| MF | GO:0048407 | | | platelet-derived growth factor binding | 3/20 | 11/17697 | 2.02e-07 | 1.88e-05 | 1.17e-05 |
| MF | GO:0019838 | | | growth factor binding | 5/20 | 137/17697 | 3.65e-07 | 1.88e-05 | 1.17e-05 |
| MF | GO:0048156 | tau protein binding | | | 3/20 | 45/17697 | 1.70e-05 | 5.83e-04 | 3.64e-04 |
| MF | GO:0099186 | structural constituent of postsynapse | | | 2/20 | 11/17697 | 6.63e-05 | 0.002 | 0.001 |
| MF | GO:0050998 | | nitric-oxide synthase binding | | 2/20 | 14/17697 | 1.10e-04 | 0.002 | 0.001 |

**TableS10. KEGG enrichment analysis.**

| ONTOLOGY | ID | Description | GeneRatio | BgRatio | pvalue | p.adjust | qvalue |
| --- | --- | --- | --- | --- | --- | --- | --- |
| KEGG | hsa04510 | Focal adhesion | 5/17 | 201/8076 | 4.41e-05 | 0.002 | 0.001 |
| KEGG | hsa05205 | Proteoglycans in cancer | 5/17 | 205/8076 | 4.84e-05 | 0.002 | 0.001 |
| KEGG | hsa04611 | Platelet activation | 4/17 | 124/8076 | 1.08e-04 | 0.004 | 0.002 |
| KEGG | hsa04530 | Tight junction | 4/17 | 169/8076 | 3.56e-04 | 0.007 | 0.004 |
| KEGG | hsa04971 | Gastric acid secretion | 3/17 | 76/8076 | 4.95e-04 | 0.007 | 0.004 |

**TableS11. GSEA analysis of mRNA dataset GSE15227.**

| ID | setSize | enrichmentScore | NES | pvalue | p.adjust |
| --- | --- | --- | --- | --- | --- |
| REACTOME_EUKARYOTIC_TRANSLATION_ELONGATION | 86 | 0.813486964 | 4.013568275 | 0.001757469 | 0.03483917 |
| REACTOME_SRP_DEPENDENT_COTRANSLATIONAL_PROTEIN_TARGETING_TO_MEMBRANE | 104 | 0.773401198 | 3.961412522 | 0.001727116 | 0.03483917 |
| KEGG_RIBOSOME | 80 | 0.805176235 | 3.939788298 | 0.001733102 | 0.03483917 |
| REACTOME_EUKARYOTIC_TRANSLATION_INITIATION | 112 | 0.750541139 | 3.92159127 | 0.001680672 | 0.03483917 |
| WP_CYTOPLASMIC_RIBOSOMAL_PROTEINS | 81 | 0.79603304 | 3.906190684 | 0.001733102 | 0.03483917 |
| REACTOME_RESPONSE_OF_EIF2AK4_GCN2_TO_AMINO_ACID_DEFICIENCY | 94 | 0.772023571 | 3.872267758 | 0.001736111 | 0.03483917 |
| REACTOME_SELENOAMINO_ACID_METABOLISM | 101 | 0.749881716 | 3.827490584 | 0.001694915 | 0.03483917 |
| REACTOME_NONSENSE_MEDIATED_DECAY_NMD_ | 108 | 0.737101219 | 3.784854787 | 0.00174216 | 0.03483917 |
| REACTOME_REGULATION_OF_EXPRESSION_OF_SLITS_AND_ROBOS | 156 | 0.656113069 | 3.614742582 | 0.001650165 | 0.03483917 |
| REACTOME_INFLUENZA_INFECTION | 145 | 0.653649203 | 3.531948812 | 0.001718213 | 0.03483917 |
| REACTOME_TRANSLATION | 263 | 0.562338742 | 3.335346439 | 0.001602564 | 0.03483917 |
| REACTOME_SIGNALING_BY_ROBO_RECEPTORS | 202 | 0.579780535 | 3.30492118 | 0.001647446 | 0.03483917 |
| REACTOME_ACTIVATION_OF_THE_MRNA_UPON_BINDING_OF_THE_CAP_BINDING_COMPLEX_AND_EIFS_AND_SUBSEQUENT_BINDING_TO_43S | 55 | 0.726870868 | 3.255004366 | 0.001886792 | 0.03483917 |
| REACTOME_RRNA_PROCESSING | 189 | 0.56880919 | 3.208404616 | 0.001647446 | 0.03483917 |
| WP_ELECTRON_TRANSPORT_CHAIN_OXPHOS_SYSTEM_IN_MITOCHONDRIA | 77 | 0.613666844 | 2.95329333 | 0.001798561 | 0.03483917 |

**TableS12.GSEA analysis of mRNA dataset GSE23130.**

| ID | setSize | enrichmentScore | NES | pvalue | p.adjust |
| --- | --- | --- | --- | --- | --- |
| WP_CYTOPLASMIC_RIBOSOMAL_PROTEINS | 80 | 0.932304587 | 2.092913662 | 0.001002004 | 0.012090739 |
| REACTOME_EUKARYOTIC_TRANSLATION_ELONGATION | 85 | 0.924719683 | 2.079966885 | 0.001002004 | 0.012090739 |
| KEGG_RIBOSOME | 79 | 0.915149437 | 2.053742423 | 0.001002004 | 0.012090739 |
| REACTOME_RESPONSE_OF_EIF2AK4_GCN2_TO_AMINO_ACID_DEFICIENCY | 93 | 0.902367605 | 2.038449275 | 0.001 | 0.012090739 |
| REACTOME_SELENOAMINO_ACID_METABOLISM | 99 | 0.896654584 | 2.028611325 | 0.000999001 | 0.012090739 |
| REACTOME_SRP_DEPENDENT_COTRANSLATIONAL_PROTEIN_TARGETING_TO_MEMBRANE | 103 | 0.891143947 | 2.015916205 | 0.001 | 0.012090739 |
| REACTOME_EUKARYOTIC_TRANSLATION_INITIATION | 111 | 0.869724405 | 1.969325472 | 0.001 | 0.012090739 |
| REACTOME_NONSENSE_MEDIATED_DECAY_NMD_ | 103 | 0.862063534 | 1.950131462 | 0.001 | 0.012090739 |
| REACTOME_REGULATION_OF_EXPRESSION_OF_SLITS_AND_ROBOS | 151 | 0.829361914 | 1.899654708 | 0.000999001 | 0.012090739 |
| REACTOME_INFLUENZA_INFECTION | 144 | 0.811577558 | 1.857750646 | 0.000999001 | 0.012090739 |
| REACTOME_ACTIVATION_OF_THE_MRNA_UPON_BINDING_OF_THE_CAP_BINDING_COMPLEX_AND_EIFS_AND_SUBSEQUENT_BINDING_TO_43S | 55 | 0.841869846 | 1.849129394 | 0.001005025 | 0.012090739 |
| WP_MIRNA_TARGETS_IN_ECM_AND_MEMBRANE_RECEPTORS | 21 | 0.891395893 | 1.839255585 | 0.001039501 | 0.012157249 |
| REACTOME_SIGNALING_BY_ROBO_RECEPTORS | 196 | 0.798434132 | 1.835323735 | 0.000999001 | 0.012090739 |
| BIOCARTA_PROTEASOME_PATHWAY | 15 | 0.93048701 | 1.828655716 | 0.001083424 | 0.012404492 |
| NABA_PROTEOGLYCANS | 34 | 0.836256363 | 1.796831005 | 0.001014199 | 0.012090739 |

**Table13. mRNA-drugs interaction network nodes.**

| gene |  | drug |  | gene |  | drug |
| --- | --- | --- | --- | --- | --- | --- |
| ACTB | - | CYCLOPHOSPHAMIDE |  | PDGFRA | - | TOVETUMAB |
| ACTB | - | ETHINYL ESTRADIOL |  | PDGFRA | - | XL-999 |
| ACTG1 | - | VINCRISTINE |  | PDGFRA | - | NINTEDANIB ESYLATE |
| COL1A1 | - | OCRIPLASMIN |  | PDGFRA | - | PAZOPANIB HYDROCHLORIDE |
| COL1A1 | - | COLLAGENASE CLOSTRIDIUM HISTOLYTICUM |  | PDGFRA | - | LENVATINIB |
| COL1A2 | - | COLLAGENASE CLOSTRIDIUM HISTOLYTICUM |  | PDGFRA | - | RIPRETINIB |
| COL1A2 | - | OCRIPLASMIN |  | PDGFRA | - | PACLITAXEL |
| PDGFRA | - | ILORASERTIB |  | PDGFRA | - | DASATINIB |
| PDGFRA | - | ENMD-2076 |  | PDGFRA | - | TAE-684 |
| PDGFRA | - | OLARATUMAB |  | PDGFRA | - | AXITINIB |
| PDGFRA | - | PAZOPANIB |  | PDGFRA | - | AST-487 |
| PDGFRA | - | NINTEDANIB |  | PDGFRA | - | CHEMBL202721 |
| PDGFRA | - | ENMD-981693 |  | PDGFRA | - | CHEMBL406845 |
| PDGFRA | - | REGORAFENIB |  | PDGFRA | - | VISTUSERTIB |
| PDGFRA | - | SU-014813 |  | PDGFRA | - | RABEPRAZOLE SODIUM |
| PDGFRA | - | TANDUTINIB |  | PDGFRA | - | TELATINIB |
| PDGFRA | - | FORETINIB |  | PDGFRA | - | CHEMBL1997335 |
| PDGFRA | - | AMUVATINIB |  | PDGFRA | - | VANDETANIB |
| PDGFRA | - | X-82 |  | PDGFRA | - | R-406 |
| PDGFRA | - | MASITINIB |  | PDGFRA | - | OSI-632 |
| PDGFRA | - | QUIZARTINIB |  | PDGFRA | - | AVAPRITINIB |
| PDGFRA | - | MOTESANIB |  | PDGFRA | - | CENISERTIB |
| PDGFRA | - | ORANTINIB |  | PDGFRA | - | CYC-116 |
| PDGFRA | - | IMATINIB |  | PDGFRA | - | ROMIPLOSTIM |
| PDGFRA | - | LUCITANIB |  | PDGFRA | - | RG-1530 |
| PDGFRA | - | SITRAVATINIB |  | PDGFRA | - | CARBOPLATIN |
| PDGFRA | - | CEDIRANIB |  | PDGFRA | - | PD-0166285 |
| PDGFRA | - | LINIFANIB |  | PDGFRA | - | AZD-1152-HQPA |
| PDGFRA | - | MIDOSTAURIN |  | PDGFRA | - | GEFITINIB |
| PDGFRA | - | PONATINIB |  | PDGFRA | - | BARASERTIB |
| PDGFRA | - | CRENOLANIB |  | PDGFRA | - | PEGPLERANIB SODIUM |
| PDGFRA | - | BECAPLERMIN |  | CLU | - | CUSTIRSEN |
| PDGFRA | - | NILOTINIB |  | CLU | - | CUSTIRSEN SODIUM |
| PDGFRA | - | RAMUCIRUMAB |  | CLU | - | LUBIPROSTONE |
| PDGFRA | - | VATALANIB |  | PTMA | - | THYMALFASIN |
| PDGFRA | - | DOVITINIB |  | PPP2CA | - | LB-100 |
| PDGFRA | - | SORAFENIB |  | C1S | - | SUTIMLIMAB |
| PDGFRA | - | SUNITINIB MALATE |  | C1S | - | CINRYZE |
| PDGFRA | - | XL-820 |  | SERPING1 | - | CINRYZE |
| PDGFRA | - | SUNITINIB |  | SERPING1 | - | RHUCIN |
| PDGFRA | - | TAK-593 |  | IGFBP5 | - | ANDROSTANOLONE |
| PDGFRA | - | FAMITINIB |  |  |  |  |

**Table14. mRNA-TF interaction network nodes.**

| mRNA |  | TF |  | mRNA |  | TF |
| --- | --- | --- | --- | --- | --- | --- |
| COL1A1 | - | CIITA |  | COL1A2 | - | RFX1 |
| COL1A1 | - | ETS1 |  | COL1A2 | - | RFX5 |
| COL1A1 | - | MKL1 |  | COL1A2 | - | SIRT1 |
| COL1A1 | - | MYB |  | COL1A2 | - | STAT6 |
| COL1A1 | - | MYBL2 |  | COL1A2 | - | YY1 |
| COL1A1 | - | NFIC |  | PDGFRA | - | CEBPD |
| COL1A1 | - | NFKB1 |  | PDGFRA | - | GLI1 |
| COL1A1 | - | RELA |  | PDGFRA | - | GLI2 |
| COL1A1 | - | SP1 |  | PDGFRA | - | PAX1 |
| COL1A1 | - | SP3 |  | HNRNPA2B1 | - | BRCA1 |
| COL1A1 | - | STAT6 |  | HNRNPA2B1 | - | MYC |
| COL1A1 | - | TFAP2A |  | CLU | - | FOS |
| COL1A2 | - | CEBPZ |  | CLU | - | FOSL1 |
| COL1A2 | - | CIITA |  | CLU | - | FOSL2 |
| COL1A2 | - | CIITA |  | CLU | - | JUNB |
| COL1A2 | - | EP300 |  | CLU | - | JUND |
| COL1A2 | - | FLI1 |  | CLU | - | MYCN |
| COL1A2 | - | HDAC1 |  | PTMA | - | ESR1 |
| COL1A2 | - | HDAC1 |  | PPP2CA | - | CREB1 |
| COL1A2 | - | HDAC2 |  | RTN3 | - | ATF6 |
| COL1A2 | - | KLF11 |  | RTN3 | - | DDIT3 |
| COL1A2 | - | MYB |  | IGFBP5 | - | ETV6 |
| COL1A2 | - | NFKB1 |  | IGFBP5 | - | TFAP2A |
| COL1A2 | - | RELA |  |  |  |  |
